# Global Identification of Multiple OsGH9 Family Members and Their Involvement in Cellulose Crystallinity Modification in Rice

**DOI:** 10.1371/journal.pone.0050171

**Published:** 2013-01-04

**Authors:** Guosheng Xie, Bo Yang, Zhengdan Xu, Fengcheng Li, Kai Guo, Mingliang Zhang, Lingqiang Wang, Weihua Zou, Yanting Wang, Liangcai Peng

**Affiliations:** 1 National Key Laboratory of Crop Genetic Improvement and National Centre of Plant Gene Research (Wuhan), Biomass and Bioenergy Research Centre, College of Plant Science and Technology, Huazhong Agricultural University, Wuhan, People's Republic of China; 2 National Key Laboratory of Crop Genetic Improvement and National Centre of Plant Gene Research (Wuhan), Biomass and Bioenergy Research Centre, College of Life Science and Technology, Huazhong Agricultural University, Wuhan, People's Republic of China; 3 National Key Laboratory of Crop Genetic Improvement and National Centre of Plant Gene Research (Wuhan), Biomass and Bioenergy Research Centre, College of Plant Science and Technology, College of Life Science and Technology, Huazhong Agricultural University, Wuhan, People's Republic of China; Lawrence Berkeley National Laboratory, United States of America

## Abstract

Plant glycoside hydrolase family 9 (GH9) comprises typical endo-β-1,4-glucanase (EGases, EC3.2.1.4). Although *GH9A* (*KORRIGAN*) family genes have been reported to be involved in cellulose biosynthesis in plants, much remains unknown about other GH9 subclasses. In this study, we observed a global gene co-expression profiling and conducted a correlation analysis between *OsGH9* and *OsCESA* among 66 tissues covering most periods of life cycles in 2 rice varieties. Our results showed that *OsGH9A3* and *B5* possessed an extremely high co-expression with *OsCESA1*, *3*, and *8* typical for cellulose biosynthesis in rice. Using two distinct rice non-GH9 mutants and wild type, we performed integrative analysis of gene expression level by qRT-PCR, cellulase activities *in situ* and *in vitro*, and lignocellulose crystallinity index (CrI) in four internodes of stem tissues. For the first time, OsGH9B1, 3, and 16 were characterized with the potential role in lignocellulose crystallinity alteration in rice, whereas OsGH9A3 and B5 were suggested for cellulose biosynthesis. In addition, phylogenetic analysis and gene co-expression comparison revealed GH9 function similarity in *Arabidopsis* and rice. Hence, the data can provide insights into GH9 function in plants and offer the potential strategy for genetic manipulation of plant cell wall using the five aforementioned novel *OsGH9* genes.

## Introduction

Cellulose is the major wall polysaccharide in plants and has a wide application for biofuel, paper, and other chemical products [1], [2]. Due to their crystalline property, cellulose microfibrils are highly recalcitrant to biomass saccharification [3]. Hence, understanding cellulose biosynthesis and crystallization is essential.

Cellulose is a fibrous polymer of glucose units linked by β-1, 4-glucosidic bonds. It can self-associate into non-crystalline and crystalline microfibrils in a plant cell wall, providing mechanical strength and flexibility during plant growth and development [4]. Over the past years, the crystallinity index (CrI) has been applied to account for lignocellulose crystallinity by characteristic X-ray diffraction (XRD) patterns and solid-state ^13^C nuclear magnetic resonance (NMR) spectra [5], [6]. In higher plants, cellulose is synthesized at the plasma membrane by a symmetrical rosette of six global protein complexes, with each complex containing several structurally similar cellulose synthase (CESA) subunits [7]. AtCESA1, 3, and 6 in *Arabidopsis* and OsCESA1, 3, and 8 in rice have been identified for primary cell wall formation, whereas AtCESA4, 7, and 8 and OsCESA4, 7, and 9 are responsible for cellulose biosynthesis in the secondary cell walls, respectively [8]–[11]. Furthermore, *AtCESA3^ixr1-2^* and *AtCESA6^ixr2-1^* mutants show increased biomass saccharification efficiency and reduced cellulose crystallinity index (CrI) [12], [13]. *CESA1^A903V^* and *CESA3^T942I^* mutants also display reduced cellulose microfibril crystallinity [14]. In addition, other genes, such as *COBRA* and *KORRIGAN*, have been reported to contribute to cellulose biosynthesis [15], [16].

Endo-β-1, 4-glucanases (EGases, EC3.2.1.4) have been found in prokaryotic and eukaryotic organisms. They are crucial for cell wall degradation and remodeling because they can cleave the internal β-1,4-glycosidic bond between two glucose moieties in the center of a polysaccharide chain [17]–[19]. Plant EGase enzymes belong to subgroup E2 of glycoside hydrolase family 9 (GH9) with three subclasses (A, B, C) [18], [20]. GH9A comprises membrane-anchored proteins [21], [22], GH9B comprises secreted proteins with only one catalytic domain (CD), and GH9C possesses a CD and a distinct C-terminal extended cellulose-binding domain (CBD) that binds to crystalline cellulose as the bacterial cellulase did [23]. Although GH9 family proteins are usually able to hydrolyze artificial soluble cellulose derivatives, such as carboxymethyl cellulose (CMC) or hydroxyethyl cellulose (HEC) [24], biochemical analyses have revealed their specificity for different substrates *in vitro* [25]–[27].

A number of *KORRIGAN* (*kor*) mutants of GH9A family genes show reduced crystalline cellulose in plants [15], [28], [29]. For instance, ectopic overexpression of *PttKOR*1 in *Arabidopsis kor1-1* mutant leads to a higher cellulose crystallinity [30], whereas RNA interference (RNAi) of *PaxgKOR* could reduce cellulose level and increase cellulose crystallinity [31]. In rice, silencing of *OsGH9A3* results in reduction of cell elongation and cellulose content; it also causes an increase of pectin content in leaves [32]. In addition, KORRIGAN protein can either cleave sterol-cellodextrin substrate [33], or remove glucan chains incorrectly assembled in the growing microfibrils [34]. Although both GH9B and GH9C have been reported with activities for cello-oligosaccharide release and xyloglucan cleavage in plants [25], [35]–[38], little is known about their functions in cellulose biosynthesis and crystallization in rice [39], [40].

Rice, an important food crop worldwide, is a model for gene function analysis in monocotyledonous plants. The completion of the rice genome sequencing may likely identify the potential function of the entire GH9 family genes in rice, based on bioinformatics analysis and related biological characterization. In this study, we initially observed a gene co-expression profiling between *OsGH9* and *OsCESA*, and then performed a comparative analysis of the multiple *OsGH9* genes' functions in cellulose synthesis and assembly, especially in their involvement in modification of lignocellulose crystallinity in rice.

## Results

### Identification of OsGH9 family in rice

**Figure 1:**
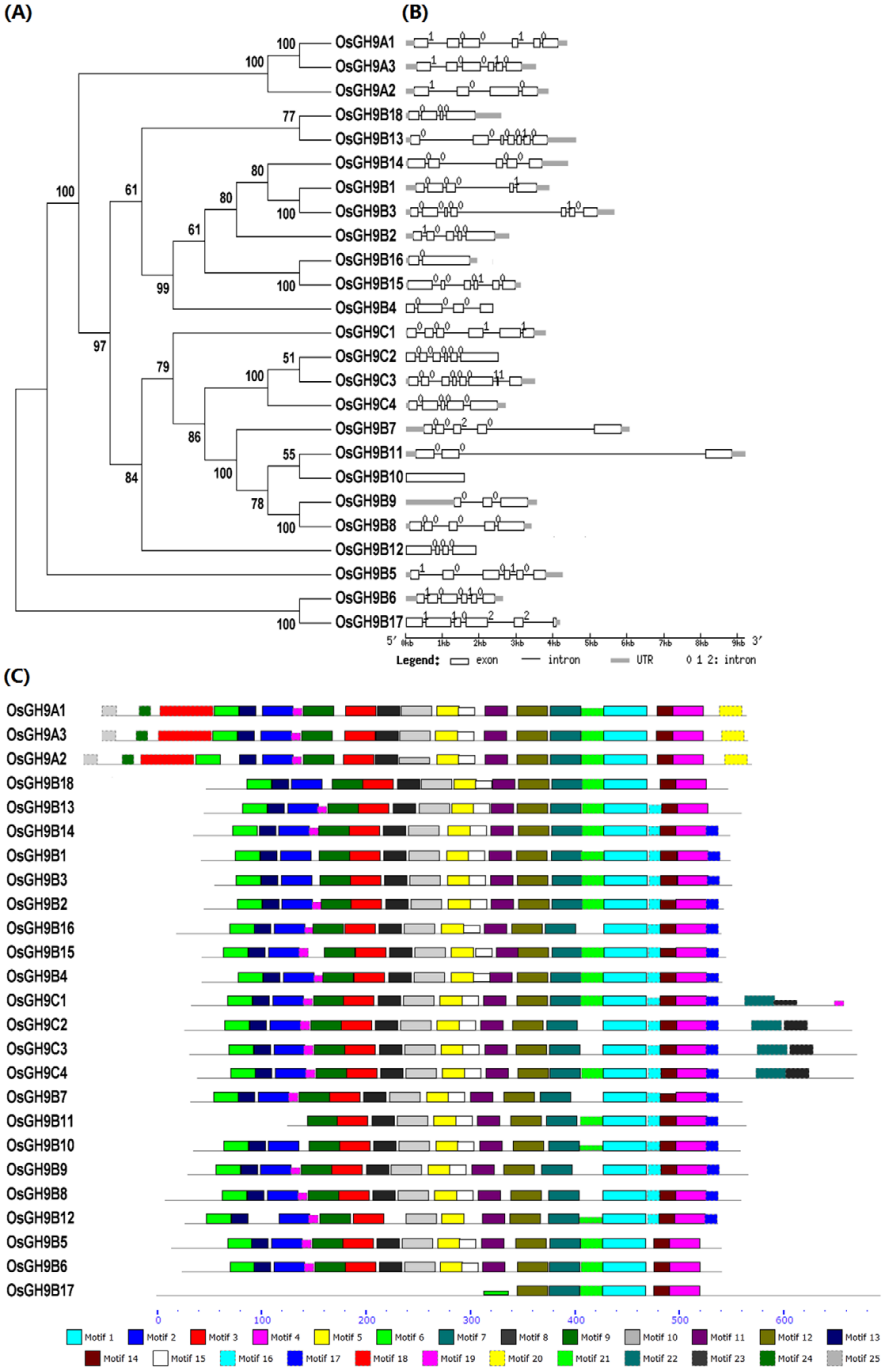
Structural classification of OsGH9 family in rice. (**A**) Phylogenetic tree of OsGH9 family proteins: Clustal X program and tree construction using MEGA3.1 used for multiple alignment analysis of 25 rice GH9 family proteins. (**B**) Exon-intron comparison of *OsGH9* family genes: GSDS http://gsds.cbi.pku.edu.cn/ was used for analysis of the exon-intron structures. (**C**) Motif distributions of OsGH9 family proteins. The 25 motifs of OsGH9 family proteins annotated in Table S8 using the interProScan search program http://www.ebi.ac.uk/Tools/InterProScan/, and then identified using the MEME program (version 4.0) http://meme.sdsc.edu/meme/cgi-bin/meme.cgi.

Searching local rice genome database yielded a total of 25 *OsGH9* family genes located in 9 chromosomes in rice, except 1 gene (LOC_Os01g64140) with a partial DNA sequence (Table S1). Based on the nomenclature of GH9 family in *Arabidopsis* [26], GH9 family proteins can be divided into three subclasses (A, B, and C) (Figure 1A). GH9A subclass (OsGH9A1, 2 and 3) contained the charged and hydrophilic amino acid-rich N-termini, transmembrane domain (TM), and proline-rich C-termini. The GH9A proteins had large tails with 71 to 73 amino acid residues (Table S1). In addition, all GH9A proteins contained polarized targeting signals (LL and YXXΦ) and six predicted glycosylation sites (data not shown). Particularly, OsGH9B18 showed the sequence homology and motif similar to OsGH9B13 (Figure 1), despite that its orthologue in *Arabidopsis* has been grouped as AtGH9A4 (Figure S4). By comparison, all GH9B and GH9C proteins did not contain any TM, but had a catalytic domain (CD) and an N-terminal cleavable signal peptide (SP) without significant sequence conservation in the extracellular secreted proteins, as shown by PSORT prediction tool (data not shown). GH9B proteins displayed diversity in the transmembrane helix and pI value, and GH9C proteins showed a putative cellulose-binding domain (CBD) as the bacterial cellulase did (Table S1).

Furthermore, there were at least 4 exons in *GH9A* and *GH9C* genes, and 1 to 7 exons in *GH9B* genes (Figure 1B). MAST analysis (http://meme.nbcr.net/) showed that GH9A1, 2, 3 and GH9C1, 2, 3, 4 contained identical and conserved motifs, whereas those in GH9B were relatively variable (Figure 1C), suggesting a diverse structural organization in GH9B family in rice.

### Co-expression profiling between *OsGH9* and *OsCESA* family

**Figure 2:**
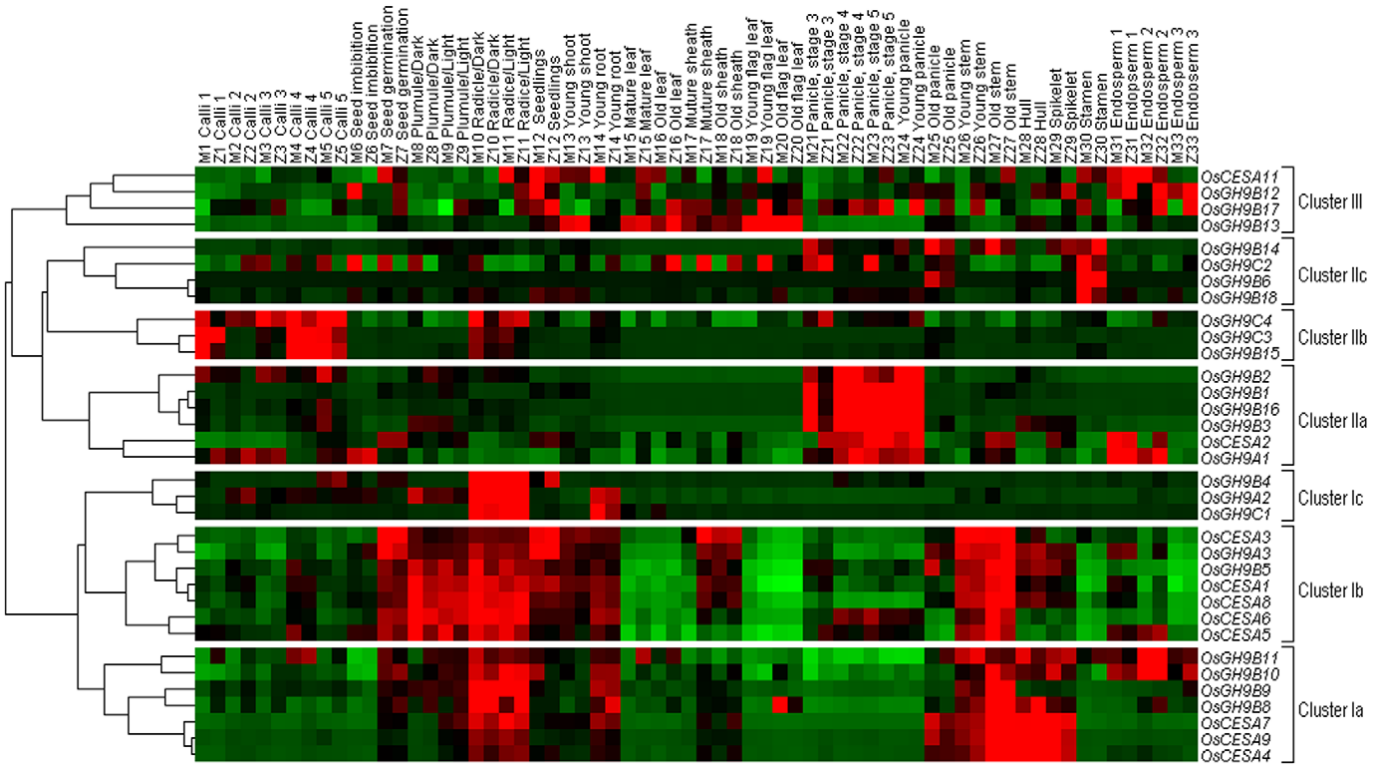
Co-expression profiling among *OsGH9* and *OsCESA* in rice. The cDNA chip data of 66 tissues obtained from two rice var. *ZS97* and *MH63* at CREP database http://crep.ncpgr.cn/cgi/home.pI website, and the transcription profiling of *OsGH9* and *OsCESA* family genes performed by the hierarchical cluster method.

As *KORRIGAN* genes have been identified to show co-expression with cellulose synthase gene *CESA* in *Arabidopsis* [41], we initially observed the co-expression profiling between entire *OsGH9* and *OsCESAs* family genes among 66 tissues (Table S9) from the most periods of life cycles of 2 rice varieties (ZS97 and MH63) using cDNA chip's CREP data (http://crep.ncpgr.cn) [42]. As a result, 25 *OsGH9* family genes can be classified into 3 expression clusters (I, II, III) with 6 subunits (Ia, Ib, Ic, IIa, IIb, IIc) (Figure 2). In general, *OsGH9B8*, *9*, *10*, and *11* genes in Cluster Ia were highly co-expressed with *OsCESA4*, *7*, and *9* typical for secondary cell wall biosynthesis [10], whereas *OsGH9A3* and *OsGH9B5* genes in Cluster Ib showed strong co-expression with *OsCESA1*, *3*, *8*, *5*, and *6* for primary cell wall formation [11]. In particular, Clusters Ic, IIa, and IIb presented a tissue-specific expression in the radicle, panicle, and calli, respectively, and Clusters IIc and III showed a weak co-expression pattern. Correlation analysis further confirmed that *OsGH9A3* and *OsGH9B5* had significant co-expression with *OsCESA1*, *3*, and *8* (*p*<0.01), suggesting that OsGH9A3 and OsGH9B5 may have a role in cellulose biosynthesis (Tables 1, S2, and S3). Notably, a significant correlation was also found among *OsGH9B1*, *2*, *3*, and *16* (Tables 2, S3), confirming their tight co-expression observed in Cluster IIa.

**Table 1:** Correlation coefficients between *OsGH9A3*/*B5* and *OsCESA1/3/8* in 66 rice tissues (n = 66).

Gene pair	*GH9A3*	*GH9B5*	*CESA1*	*CESA3*	*CESA8*
*GH9A3*	1	0.821**	0.866**	0.880**	0.875**
*GH9B5*		1	0.891**	0.739**	0.859**
*CESA1*			1	0.835**	0.951**
*CESA3*				1	0.853**
*CESA8*					1

** , significant test at *p*<0.01. Total of 66 data (n = 66) obtained from the cDNA chip data of 33 tissues of two rice varieties (*ZS97* and *MH63*) at CREP database http://crep.ncpgr.cn/crep-cgi/home.pl as shown in Figure 2. Method of the correlation analysis shown in “Materials and Methods”.

**Table 2:** Correlation coefficient among *OsGH9B1*, *2*, *3* and *B16* in 66 rice tissues (n = 66).

Gene pair	*GH9B1*	*GH9B2*	*GH9B3*	*GH9B16*
*GH9B1*	1	0.790**	0.806**	0.761**
*GH9B2*		1	0.879**	0.916**
*GH9B3*			1	0.855**
*GH9B16*				1

** , significant test at *p*<0.01. Total of 66 data (n = 66) obtained from the cDNA chip data of 33 tissues of two rice varieties (*ZS97* and *MH63*) each at CREP database http://crep.ncpgr.cn/cgi/home.pI as shown in Figure 2; Method of the correlation analysis shown in “Materials and Methods”.

Furthermore, 18 *GH9* family gene expressions were detected by qRT-PCR (Figure S1). Both *OsGH9A3*, *B5*, and *OsCESA1*, *3*, *8* were highly expressed in radicle, plumule, and internodes tissues. Although *OsGH9B1*, *3*, and *16* genes were determined with high expression levels, *OsGH9B2* gene was undetectable by qRT-PCR in most tissues. In addition, *OsGH9A2* and *OsGH9C1* showed the specifically high expression in the radicle tissues. *OsGH9B6* was highly expressed in stamen tissues, and other *OsGH9* family genes were expressed in several tissues in rice.

### Analysis of two rice mutants

**Figure 3:**
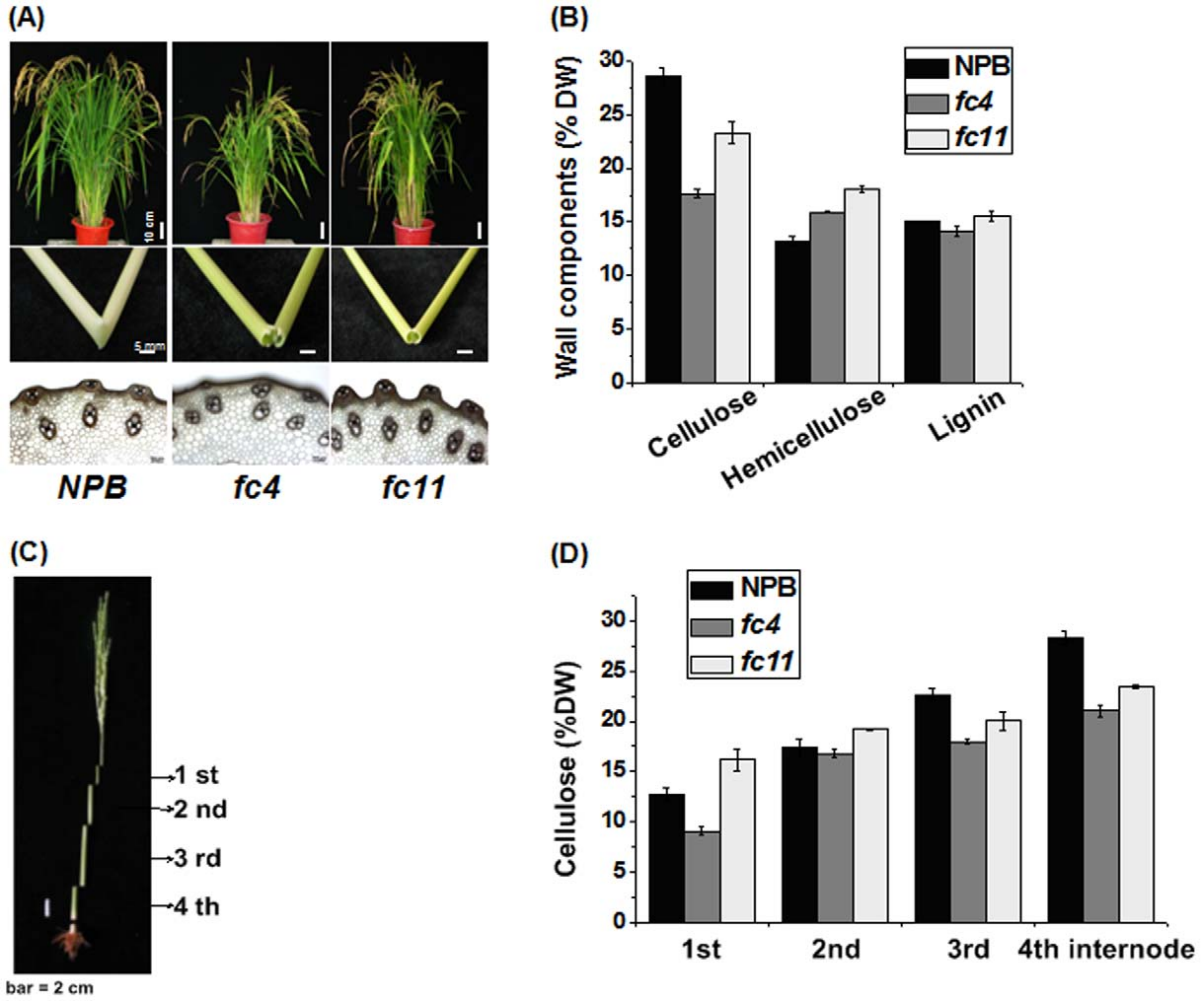
Morphological observation and carbohydrate analysis of two rice mutants. (**A**) Phenotypes of two rice mutants (*Osfc4*, *Osfc11*) and wild type (*NPB*): upper panel, rice growth at mature stage; middle panel, manual bending of mature culms; down panel, stem dissection under light microscope. (**B**) Cell wall composition of mutants and wild type in the mature stems: Cellulose, hemicelluloses and lignin (% DW, dry weight) contents expressed as means ± SD (n = 3). (**C**) Diagram of four internodes at booting stage. (**D**) Cellulose contents (% DW, dry weight) of four internodes as shown in (C): Data as means ± SD (n = 3).

After large-scale screening and identification of rice mutants in morphological phenotypes and cell wall characteristics including cell wall components, compositions and cell wall degradability, we selected two distinct T-DNA (*Osfc4* and *Osfc11*) mutants with genetic backgrounds of Nipponbare (NPB, wild type) in order to identify GH9 family's potential function on cellulose biosynthesis and crystallization (Figure 3). Both homozygous mutants exhibited a normal growth phenotype during most of their life cycles. However, both were detected with fragile culms compared with the wild type (Figure 3A). Carbohydrate analysis indicated that the two mutants had much lower cellulose and relatively higher hemicellulose levels than wild type in their mature stem tissues (Figure 3B). The stem tissues from 1^st^ to 4^th^ internodes at booting stage of rice presented a consistent increasing course of cellulose biosynthesis, so we observed a typical alteration of cellulose content among mutants and wild type (Figures 3C and 3D). However, the two mutants and the wild type showed a large difference in cellulose production in 1^st^ and 4^th^ internodes, providing the experimental materials excellent for functional analysis of GH9 family genes [43]. In addition, both mutants were genetically identified to be the non-GH9 genes involved in cell wall biosynthesis and modification (data not shown), which allowed the functional analysis of entire OsGH9 family genes in rice.

### Correlation among cellulase activity, lignocellulose crystallinity, and *OsGH9* RNA transcripts

**Figure 4:**
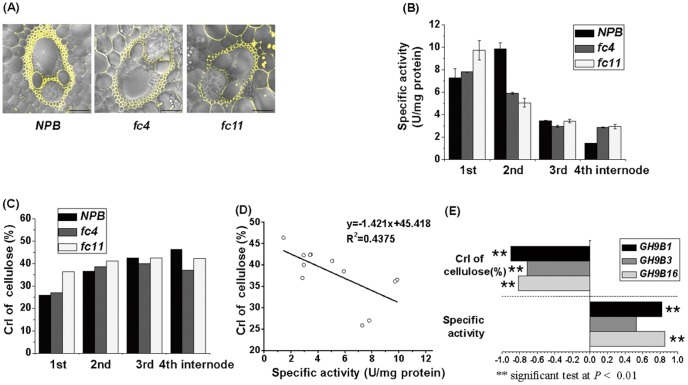
Correlation analysis among cellulase activity, lignocellulose crystallinity index and *OsGH9* gene expression in four internodes of mutants (*fc4* and *fc11*) and wild type (*NPB*) at booting stage. (**A**) Observation of cellulase activity *in situ* of the 3^rd^ internode tissues: The detection of cellulase activity *in situ*, and the bar indicated 50 µm. (**B**) Assay of *in vitro* cellulase specific activity (U. mg^−1^ protein) in four internodes tissues: Total proteins of four different internodes were used for *in vitro* cellulase assay by Markergene fluorescent Cellulase Assay Kit. (**C**) Lignocellulose crystallinity index (CrI) of four internodes tissues. (**D**) Correlation (n = 12) between cellulase specific activity and lignocellulose CrI among 12 internode samples of four stems in mutants (*fc4* and *fc11*) and wild type (*NPB*). (**E**) Correlation coefficients (n = 12) between *OsGH9B1*, *3*, and *16* transcript levels and cellulase specific activity or lignocellulose CrI.

Using two rice mutants and wild type, we initially detected cellulase activity *in situ* and *in vitro* in the stem internode tissues at booting stage. As shown in Figure 4A, the cellulase activity *in situ* could be observed in the cell walls of vascular bundles of internodes in the stem tissues. The cellulase activity *in vitro* with substrate resorufin cellobioside was quantitatively determined with a dynamic alteration during stem internode growth and development (Figures 4B and S2). As a result, both mutants and wild type displayed much higher cellulase activities in young internode (1^st^ and 2^nd^) tissues than in the old ones (3^rd^ and 4^th^), indicating cellulase predominant activity in primary cell wall biosynthesis. Meanwhile, we detected lignocellulose crystallinity in all tissues with a consistent increase CrI value from 1^st^ to 4^th^ internodes in both mutants and wild type (Figure 4C). Furthermore, a correlation analysis was conducted between cellulase activity and lignocellulose CrI with *R^2^* value at 0.44 (Figure 4D). Despite the *R^2^* value being less than 0.5, the correlation coefficient value significantly reached −0.70 (*p*<0.05), suggesting that cellulase may modify cellulose crystallinity.

We then analyzed the representative gene expression levels of 3 clusters (Ia, Ib, and IIa) in 4 internode tissues of mutants and wild type by qRT-PCR analysis (Figure S1, Table S4) to test the GH9 family genes' involved roles in the alteration of lignocellulose crystallinity. *GH9B1* and *GH9B16* in Cluster IIa showed extremely high correlation coefficient values (0.813 to 0.902, at *p*<0.01, respectively), either positively with cellulase specific activity or negatively with lignocellulose CrI (Figure 4E and Table S5). Although *GH9B3* in Cluster IIa did not display a significant correlation with cellulase activity, its coefficient value related to lignocellulose CrI reached −0.716 at *p*<0.01. Hence, our findings suggest that OsGH9B1, B3, and B16 may have enzymatic activities for lignocellulose crystallinity modification in rice in terms of stem internode growth and development. By contrast, other *GH9* genes in Clusters Ia and Ib did not show any significant correlation with lignocellulose CrI or cellulase activity.

Furthermore, *OsGH9A3* and *OsGH9B5* exhibited significantly positive co-expression (*p<0.01*) with *OsCESA1*, *3*, and *8* among the four stem internodes of mutants and wild type, but other *OsGH9B* genes did not show any positive co-expression (Tables S4, S6). Notably, *OsGH9A1* was highly co-expressed with *OsCESA3*, *4*, *8* (*p<0.05*), whereas *OsGH9B1* and *OsGH9B16* were negative with *OsCESA7* (*p<0.01*). It was confirmed that *OsGH9A3* and *B5*, other than *OsGH9B1*, *3*, *16*, may play a role in cellulose biosynthesis, although additional evidences need to provide. It also suggests that *OsGH9A1* may have an effect on cellulose biosynthesis at least in the rice stem internode growth and development.

### Comparison with AtGH9 family in *Arabidopsis*

According to the catalytic domain (CD) analysis, 25 putative AtGH9 family genes with 3 subclasses (A, B, and C) can be found in *Arabidopsis* [26] Based on the co-expression profiling among the 63 tissues in *Arabidopsis*, 25 *AtGH9* genes can also be classified into 3 clusters (Figure S3, Table S10). By comparison, *AtGH9A1* and *B7* in Cluster Ic, like *OsGH9A3* and *B5* in Cluster Ib, were highly co-expressed with *AtCESA1*, *3*, and *6* typical for primary cell wall biosynthesis in *Arabidopsis* (Table 3). Furthermore, *AtGH9B1* and *B2* showed high co-expression in the flower and carpel tissues, similar to *OsGH9B1*, *B2*, *B3*, and *B16* expression in panicle tissue. In addition, phylogenetic analysis was consistent with the co-expression patterns in rice and *Arabidopsis* (Figure S4). Due to the close relationship in the phylogenetic tree, *AtGH9A1* and *B7* were suggested to have a role in cellulose biosynthesis, whereas *AtGH9B1* and *B2*, like *OsGH9B1*, *3*, *16* in rice, may have enzymatic activities for lignocellulose crystallinity modification in *Arabidopsis*.

**Table 3:** Comparison of gene functional patterns in rice and *Arabidopsis* based on the co-expression profiling data in Figure 2 and Figure S3.

Cluster	Rice		Cluster	Arabidopsis	
	Tissues	Genes		Tissues	Genes
Ia	radicle, stem, hull	*CESA4/7/9*, *GH9B8/9*	Ia	root, siliques	*CESA4/7/8*, *GH9B8*
Ib	radicle, plumule, seedlings, stem	*CESA1/3/8/5/6*, *GH9A3*, *GH9B5*	Ic	root, leaf, seedlings, whole plant	*CESA1/3/6/2/5*, *GH9A1*, *GH9B7*
Ic	radicle	*GH9A2*, *GH9B4*, *GH9C1*	II	radicle	*GH9A2*, *GH9B3/4*, *GH9C1*, *GH9C3*
IIa	panicle	*GH9B1/2/3/16*	Ib	shoot apex, flowers, carpels	*GH9B1/2*
IIc	stamen	*GH9B18*, *GH9B6/14*, *GH9C2*	III	stamen, pollen	*GH9A3*, *GH9A4*, *GH9B5/11/14/17*

The co-expression data of rice and *Arabidopsis* GH9 family genes derived from Figure 2 and Figure S3.

## Discussion

Large-scale co-expression has been performed to identify the gene functions in plant cell wall formation across plant species [44]. In *Arabidopsis*, *KORRIGAN* mutants have been characterized as GH9A family genes involved in cellulose biosynthesis [15], [28]–[30]. The regression analysis of the 408 publicly available microarray data sets could even reveal the *AtKOR1* (*AtGH9A1*) co-expression with *AtCESA1*, *3*, and *6* typical for primary cell wall biosynthesis [41]. In aspen tree, RT-PCR, *in situ* hybridization, and tissue-print assays demonstrated the co-expression of *PtrKOR* with *PtrCESA1*, *2*, and *3* genes associated with the secondary cell wall synthesis in xylem cells [45]. Although several *GH9A* (*KOR*) genes have been identified in *Arabidopsis*, rice, and other plants [32], [46], little remains known about other OsGH9 subclass functions. In this study, global co-expression profiling and correlation analysis indicated that two subclasses of *OsGH9* family genes (*OsGH9A3* and *OsGH9B5*) were highly co-expressed with *OsCESA1*, *3*, and *8* genes typical for cellulose synthesis in rice. Similarly, two subclasses of *AtGH9* family genes (*AtGH9A1* and *AtGH9B7*) were also identified for cellulose biosynthesis in *Arabidopsis*. It suggests that genetic silencing of *OsGH9A3* or *OsGH9B5* or both genes in the transgenic plants may clarify their potential role, like *KORRIGAN*, in cellulose biosynthesis in rice and other plants. More importantly, co-expression patterns of the genes could somewhat suggest the potential interaction or coordination of their proteins. For instance, based on the high co-expression pattern, *OsGH9B1*, *3*, and *16* were initially suggested to be involved in lignocellulose crystallinity modification, other than in cellulose biosynthesis, which was sequentially confirmed by integrative analysis among gene expression level, cellulase activity, and lignocellulose CrI. Obviously, gene co-expression profiling and correlation analysis are powerful tools in the identification of the entire GH9 family genes' functions in rice and other plants.

The *in situ* hybridization of endo-β-1, 4-glucanase in differential tissues has been reported in pine [47], *Brassica napus* [48], and aspen trees [45]. A novel, real-time fluorogenic assay with resorufin-β-cellobioside as a substrate has been used for observing glycoside hydrolase activity in *planta*. Recently, the resorufin glycosides have been detected with high sensitivity *in muro* cellulase enzyme activity assay due to the significant resorufin ionization at typical apoplastic pH values [49]. By this means, *KOR1-*overexpressing *Arabidopsis* plants have been found to show increased cellulase activity in stem tissues compared with wild-type plants [30]. In this study, the cellulase activity *in situ* was observed specifically in the cell wall of the four internode stems in rice, and the cellulase activities *in vitro* were quantified and compared between mutants and wild type. Notably, both wild type and mutants displayed a consistent increase at cellulose levels and CrI values from 1^st^ to 4^th^ internode, but showed a constant decrease at cellulase activity *in vitro* except at 2^nd^ internode of wild type. It suggests that the four internodes would be model materials accounting for cellulase effect on cellulose biosynthesis and lignocellulose crystalline feature in rice and other plants. Despite two mutants and wild type showed a difference at each internode, we could conduct a correlation analysis using all four internodes of wild type and mutants. The correlation calculations among *OsGH9* expression level, cellulase activity, and lignocellulose crystallinity, could indicate that OsGH9B1, 3, and 16 have specific cellulase activities for lignocellulose crystallinity modification, other than for cellulose biosynthesis. By contrast, *OsGH9A3* and *B5* did not show any significant correlation either with cellulase activity or lignocellulose CrI. Hence, we could interpret that the changed lignocellulose crystallinity of *AtGH9A1* (*Kor*) may be a consequence of cellulose biosynthesis, because recent report has indicated that CesA mutant could result in the lignocellulose crystallinity alteration [58].

Mutant selection and reverse genetic analysis have been broadly applied to identify the target gene function in plants. However, both approaches have their typical limitations and disadvantages, especially if the target gene is lethal to plant growth, or genetically redundant, or functionally dependent on isoform coordination [13], [49], [50]. Alternatively, we found that analysis of non-GH9 rice mutants is a useful approach for identification of the multiple *OsGH9* family genes. Because the two rice mutants used in this study were genetically identified as the non-GH9 mutants (data not shown), we could conduct a correlation analysis among all detectable *OsGH9* gene expressions, cellulase activity, and lignocellulose crystallinity in the wild type and two mutants, suggesting that OsGH9B1, 3, and 16 have a coordinate function on lignocellulose crystallinity modification. To our knowledge, the GH9B subclass gene function in plants has not yet been discovered. Although the GH9B function could be investigated by reverse genetic analysis, the extremely high co-expression and coordination among *OsGH9B1*, *3*, and *16* suggest that silencing of the individual OsGH9B isoform gene may not result in any significant alteration of lignocellulose crystallinity. In this case, co-silencing of *OsGH9B1*, *3*, and *16* in a mutant may be essential for functional analysis in the future. Hence, non-GH9 mutants are advantageous for function analysis of the entire OsGH9 family genes.

A total of 25 GH9 family genes with 3 subclasses (A, B, and C) were identified in both rice and *Arabidopsis*. GH9A (KORRIGAN) proteins, such as OsGH9A3 and AtGH9A1, containing the transmembrane domain, could be co-localized with CESA complex [51], indicating their involvement in cellulose biosynthesis. However, GH9B subclass, such as OsGH9B5 and AtGH9B7, comprised the secreted proteins, suggesting that GH9B should be distinguished from GH9A for cellulose biosynthesis. OsGH9B1/2/3/16 and AtGH9B1/2 were proposed to have the enzymatic activity for lignocellulose crystallinity alteration, other than for cellulose biosynthesis, indicating that these proteins may have specific activities for post-modification of cellulose microfibers in the cell wall. In addition, GH9C subclass comprised the secreted proteins, but had a C-terminal CBD to crystalline cellulose, suggesting a specific role in the turnover of crystalline cellulose [23], [26]. Although GH9C function was not identified in this study, *OsGH9C1* and *AtGH9C1/3* were highly and specifically expressed in the radicle tissues, providing potential for further investigation in the future.

## Conclusions

Global gene co-expression profiling and correlation analysis based on microarray data from 66 tissues of 2 rice varieties indicated the OsGHA3 and B5 potential role in cellulose biosynthesis. Integrative analysis of OsGH9 gene expression level, cellulase specific activities *in situ* and *in vitro*, and lignocellulose crystallinity (CrI) in distinct two rice mutants and wild type revealed that OsGH9B1, 3, and 16 may have enzymatic activities for lignocellulose crystallinity modification. The results can provide new insights into OsGH9 function in plants and offer a strategy for genetic manipulation of OsGH9 genes toward bioenergy crop breeding in rice.

## Materials and Methods

### Plant materials and growth condition

A japonica rice var. *Nipponbare* (*NPB*) and T-DNA insertion homozygous mutant lines (*Osfc4*, *Osfc11*) were grown in the field of Huazhong Agricultural University, Wuhan, China. When the tip of panicle just protruded out of the flag leaf at booting stage, four different internodes of stems were collected to analyze the OsGH9 family gene expression, *in situ* and *in vitro* cellulase activities and cell wall component determination. When the panicles protruded out 2 cm above the top of the flag leaf, stems were sampled for the analysis of qRT-PCR and cell wall component. All tissues were obtained from 3–6 plants and pooled for each biological replicate in the biological triplicate.

### Phylogenetic and structural analysis

The Hidden Markov Model (HMM) profile of the glycoside hydrolase family 9 domain (PF00759.1) was downloaded from PFam http://pfam.sanger.ac.uk/. We employed a name search and the protein family ID PF00759.1 for the identification of OsGH9 genes from the rice genome. Information about the chromosomal localization, coding sequence (CDS), amino acid (AA) and full length cDNA accessions was obtained from TIGR http://www.tigr.org and KOME http://cdna01.dna.affrc.go.jp/cDNA. The multiple alignment analysis with counterparts in *Arabidopsis* was performed using the Clustal X program (version 1.83) [52], the unrooted phylogenetic trees were constructed with the MEGA3.1 program [53] and the neighbor joining method with 1,000 bootstrap replicates.

Motif of protein sequences were analyzed and identified using the MEME program (version 4.0) http://meme.sdsc.edu/meme/cgi-bin/meme.cgi with the following parameters: number of repetitions, any; maximum number of motifs, 25; optimum motif width set to >6 and <200. The motifs were annotated using the InterProScan http://www.ebi.ac.uk/Tools/InterProScan/search_program. The exon-intron structure analysis was performed using GSDS http://gsds.cbi.pku.edu.cn/ [54]. The protein transmembrane helices were predicted by the TMHMM Server V2.0 http://www.cbs.dtu.dk/services/TMHMM/ [55].

### Co-expression profiling analysis

The transcriptional profile data of GH9 family and CESA family genes in 33 tissues (Table S9) of *Zhenshan 97* (*ZS97*) and *Minghui 63* (*MH63*), Z1–Z33 and M1–M33 (Figure 2) was respectively obtained from the CREP database http://crep.ncpgr.cn. Massively parallel signature sequencing (MPSS) data website: http://mpss.udel.edu/rice/mpss_index.php [56] was used to determine the transcriptional profiles of the genes with conflicting probe set signals. The expression values were log-transformed, and cluster analysis was performed using a software cluster with Euclidean distances and the hierarchical cluster method of complete linkage clustering as described in [11], [42]. Correlation coefficients of these gene expressions were also calculated to determine whether they are significantly different at 0.01 or 0.05 percent levels, respectively

The gene expression profiling of *AtGH9* and *AtCES*A families in the 66 tissues of Arabidopsis (Table S10) was based on the Gene Expression Omnibus database http://www.ncbi.nlm.nih.gov/geo/ using the GSE series accession numbers GSE5629, GSE5630, GSE5631, GSE5632, GSE5633 and GSE5634. The raw data were processed with the Affymetrix Microarray Analysis Suite (MAS Version 5, Affymetrix) [57]. Subsequent analysis of the gene expression data was performed in the statistical computing language R http://www.r-project.org using packages available from the Bioconductor project http://www.bioconductor.org as described in [11].

### qRT-PCR analysis

Total RNA was isolated from samples using RNAprep pure Plant Kit (DP432, TIANGEN BIOTECH), and 5 µg total RNA was reverse transcribed with an oligo(dT)_18_ primer in a 50 µl reaction using an M-MLV Reverse Transcriptase (Promega, USA) according to the manufacturer's instructions. The qRT-PCR was performed in a 20 µl reaction system (cDNA template 2.0 µl, 2×SYBR Green1 Mix10 µl, primer-F 0.5 µl, primer-R 0.5 µl, MilliQ 7.0 µl) with SYBR Green qPCR kit (ZOMANBIO, China) on Two Color Real-time PCR Detection System (MyiQ2, BIO-RAD) using the following program: 2 min at 95°C followed by 40 cycles of 15 sec at 95°C, 15 sec at 60°C, 25 sec at 72°C. *Ubiquitin* gene (AK059011) was used as an internal standard in the qRT-PCR. The gene expression unit was subjective to the percentage of the target gene expression value relative to the internal standard (*Ubiquitin* gene). All quantitative PCR experiments were performed in biological triplicate. All the gene-specific primers used were listed in Table S7.

### *In situ* and *in vitro* cellulase activity assay

*In situ* cellulase assay was performed as described previously by Takahashi et al [30] with minor modifications. The 3^rd^ internodes of rice stems were hand-sectioned (≈100 µm) and placed in 0.1 M MES buffer (pH 6.0). Tissue sections were transferred to 0.1 mM Resorufin Cellobioside (Markergene™ Fluorescent Cellulase Assay Kit, Product M1245, USA) in 0.1 M MES buffer (pH 6.0) for 30 min, and then the fluorescence intensity was detected by time-lapse confocal laser scanning microscopy (Carl Zeiss, LSM 510 META) equipped with a green fluorescent filter (excitation 567 nm, emission 595 nm). The same scanning parameters were used for all samples.

*In vitro* cellulase activity was also detected by Markergene™ Fluorescent Cellulase Assay Kit. Four internodes of rice stems with 0.1 g each were ground to powder in liquid nitrogen, and suspended in 300 µl reaction buffer (100 mM sodium acetate buffer, pH 6.0) at 4°C for 5 min. The supernatant was collected after centrifuge twice at 18,000 *g* at 4°C for 15 min (Eppendorf Centrifuge 5417 R). 50 µl of supernatant was incubated with 50 µl of 0.5 mM Resorufin Cellobioside (substrate reagent) and reacted for 5 min in a black flat-bottomed 96-well microtiter plate (Greiner Microlon).

Fluorescence intensity of resorufin released was measured with excitation (550 nm) and emission (595 nm), at 35°C for 45 cycles with a cycle time of 1 min using Multimode microplate reader (TECAN Infinite M200) according to the method described by Takahashi J et al [30]. Fluorescence values of blank (50 µl substrate reagent was added to 50 µl reaction buffer) were subtracted at each time point. A standard curve of Resorufin ranging from 0 to 50 µM was prepared to determine concentration of Resorufin in rice internode tissues extracts reacted with Resorufin Cellobioside. The protein concentration of samples was measured by Bradford method in triplicate. All the reactions were performed with biological triplicates.

### Plant cell wall fractionation and wall polysaccharide analysis

The plant tissues, including internodes or stems of different stages, were first heated at 105°C for 20 min, dried to constant weight at 60°C for about 7 d and kept dry until use. The extraction and fractionation of cell wall polysaccharides were performed as previously described by Peng et al with minor modification [58]. The crude cell wall material was extracted with 0.5% (w/v) ammonium oxalate and heated for 1 h in a boiling water bath. The remaining pellet was suspended in 4 M KOH containing 1.0 mg.ml^−1^ sodium borohydride for 1 h at 25°C, and the combined supernatant was neutralized, dialyzed and lyophilized for total hemicelluloses analysis. The KOH non-extractable residue was further extracted with acetic-nitric acids for 1 h at 100°C and the remaining pellet was used for cellulose determination. All samples were carried out in biological triplicate.

Colorimetric assay of total hexoses and pentoses: UV–VIS Spectrometer (V-1100D, Shanghai MAPADA Instruments Co., Ltd. Shanghai, China) was used for the absorbance reading. Hexoses were detected using the anthrone/H_2_SO_4_ method, and pentoses were detected using the orcinol/HCl method. For cellulose determinations, the cellulose sample was dissolved in 67% (v/v) H_2_SO_4_ (1.0 ml) with shaking at 25°C for 1 h, and then 10.0 µl aliquot was used for the anthrone/H_2_SO_4_ method. Total hemicelluloses level was subject to the sum total of hexoses and pentoses. Considering that high pentoses level can affect the absorbance reading at 620 nm for hexoses content by anthrone/H_2_SO_4_ method, the deduction from pentoses reading at 660 nm was carried out for final hexoses calculation. A series of xylose concentrations were analyzed for plotting the standard curve referred for the deduction, which was verified by GC-MS analysis. All experiments were carried out in biological triplicate.

Total lignin content was determined by two-step acid hydrolysis method. The solution was filtered with membrane filter (0.22 µm). 20 µl solution was injected into a HPLC (Waters 1525 HPLC) column Kromat Universil C18 (4.6 mm×250 mm, 5 µm) operating at 28°C with CH_3_OH∶H_2_O∶HAc (25∶74∶1, v/v/v) carrier liquid at the flow rate of 1 ml.min^−1^. All experiments were carried out in biological triplicate.

### Detection of the crystallinity index

Detection of crystallinity index of lignocellulose (CrI) was described by Xu et al [59] with a minor modification. The internodes of rice stem tissues at booting stages were cut into small pieces through 40 mesh sieve. The fine raw biomass powder of the plant tissue was laid on the glass sample holder (35×50×5 mm), analyzed under plateau conditions: Ni-filtered Cu Kα radiation (λ = 0.154056 nm) generated at 40 kV and 18 mA, and scanned at speed (0.0197°/s ) from 10° to 45°. CrI was calculated as 100×(I200−Iam)/I200, where I200 is the intensity of the 200 peak (θ = 22.5°), Iam is the intensity at the minimum between the 200 and 110 peaks (θ = 18.5°). Standard error was detected at ± 0.05∼0.15 using five samples in triplicate.

### Statistic calculation

The SPSS 17.0 was used for statistical analysis. Correlation coefficients were generated by performing Spearman rank correlation analysis for all pairs of measured traits across the whole population. This analysis used average values calculated from all original determinations for a given traits pair.

## Supporting Information

Figure S1 Representative *OsGH9* and *OsCESA* gene expression pattern by qRT-PCR in six tissues in rice variety (*NPB*). Eighteen representative *OsGH9* family genes and six *OsCESA* genes (*OsCESA4*, *7* and *9*, *OsCESA1*, *3* and *8*) detected by qRT-PCR in six tissues of japonica rice variety *Nipponbare* (*NPB*).
(TIF)

Figure S2 Time course of *in vitro* cellulase specific activities in four internodes of mutants (*fc4* and *fc12*) and wild type (*NPB*) at booting stage. Total proteins used for *in vitro* cellulase activity using the Resorufin Cellobioside as substrate in the time-course of 45 cycles with a cycle time of 1 min each at 35°C.
(TIF)

Figure S3 Co-expression profiling among *AtGH9* and *AtCESA* family genes in *Arabidopsis*. The expression profiling of *AtGH9* and *AtCES*A family genes based on *Arabidopsis* microarray data GSE5629, GSE5630, GSE5631, GSE5632, GSE5633 and GSE5634, and performed by the hierarchical cluster analysis.
(TIF)

Figure S4 Phylogenetic comparison of GH9 families in rice and *Arabidopsis*. The sequences of GH9 family proteins obtained from rice (http://rice.plantbiology.msu.edu/) and *Arabidopsis* (http://www.arabidopsis.org/) were aligned with Clustal X program and then constructed a phylogenetic tree using MEGA3.1 software.
(TIF)

Table S1 Information of 25 *OsGH9* genes in rice.
(DOCX)

Table S2 Correlation coefficients between *OsGH9* and *OsCESA* expression levels in 66 tissues of *ZS97* and *MH63* (n = 66).
(DOCX)

Table S3 Correlation coefficients among *OsGH9* expression levels in 66 tissues of *ZS97* and *MH63* (n = 66).
(DOCX)

Table S4 Transcript levels (%) changes of *OsGH9* and *OsCESA* in four internodes of mutants (*fc4* and *fc11*) and wild type (*NPB*) at booting stages.
(DOCX)

Table S5 Correlation coefficients between *OsGH9* genes expression level and cellulase specific activity or lignocellulose CrI in 12 internodes of mutants (*fc4* and *fc11*) and wild type (*NPB*) at booting stages (n = 12).
(DOCX)

Table S6 Correlation coefficients between *OsGH9* and *OsCESA* expression levels in rice at booting stage (n = 12).
(DOCX)

Table S7 Primer pairs of genes for qRT-PCR.
(DOCX)

Table S8 List of 25 putative motifs in the OsGH9 family proteins.
(DOCX)

Table S9 Tissue samples from 33 developmental stages of two rice varieties.
(DOCX)

Table S10 Tissue samples from 63 different developmental stages in *Arabidopsis*.
(DOCX)
