# Supplementary figures and images for: Global Identification of Multiple OsGH9 Family Members and Their Involvement in Cellulose Crystallinity Modification in Rice
Source: PLoS One. 2013 Jan 4;8(1):e50171. doi: 10.1371/journal.pone.0050171 (PMC3537678; doi:10.1371/journal.pone.0050171)

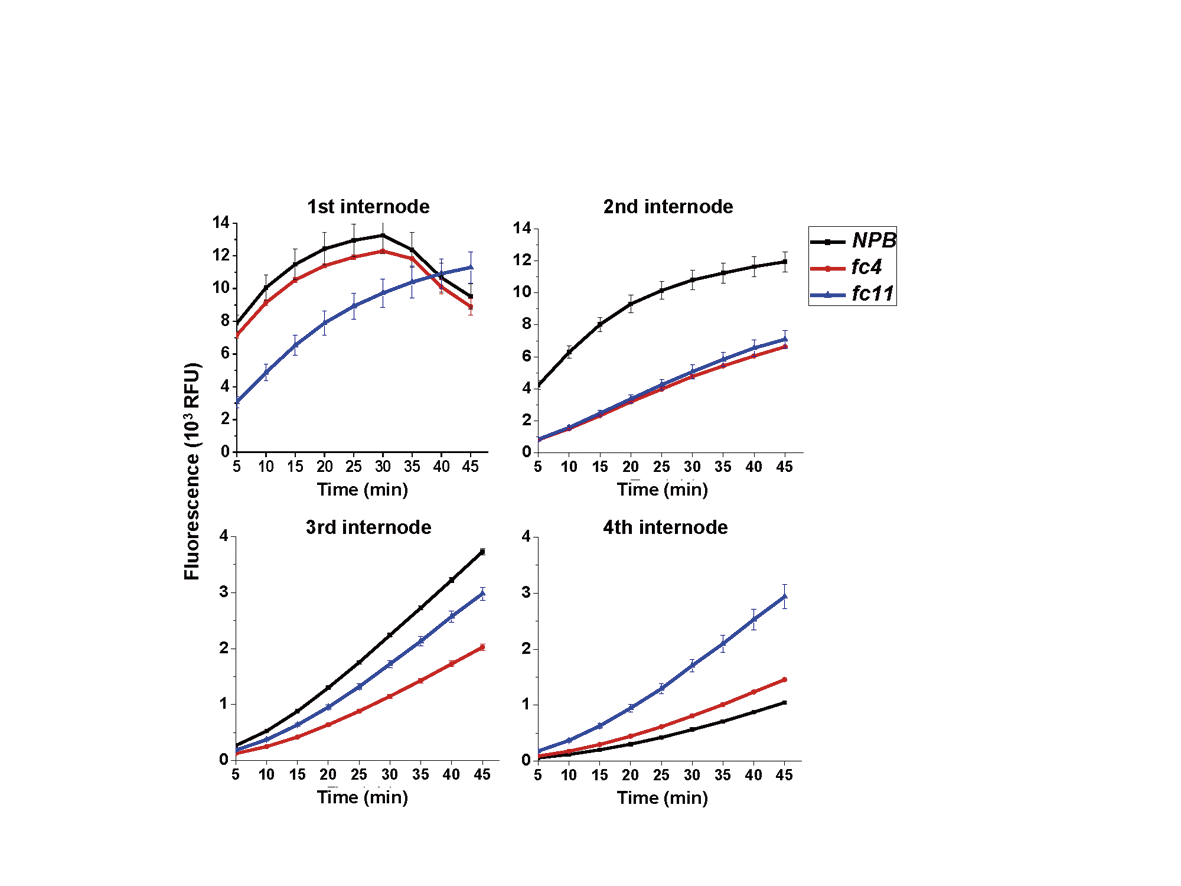

Supplement: Figure S2 — Time course of in vitro cellulase specific activities in four internodes of mutants (fc4 and fc12) and wild type (NPB) at booting stage. Total proteins used for in vitro cellulase activity using the Resorufin Cellobioside as substrate in the time-course of 45 cycles with a cycle time of 1 min each at 35°C. (TIF) [file pone.0050171.s002.tif]

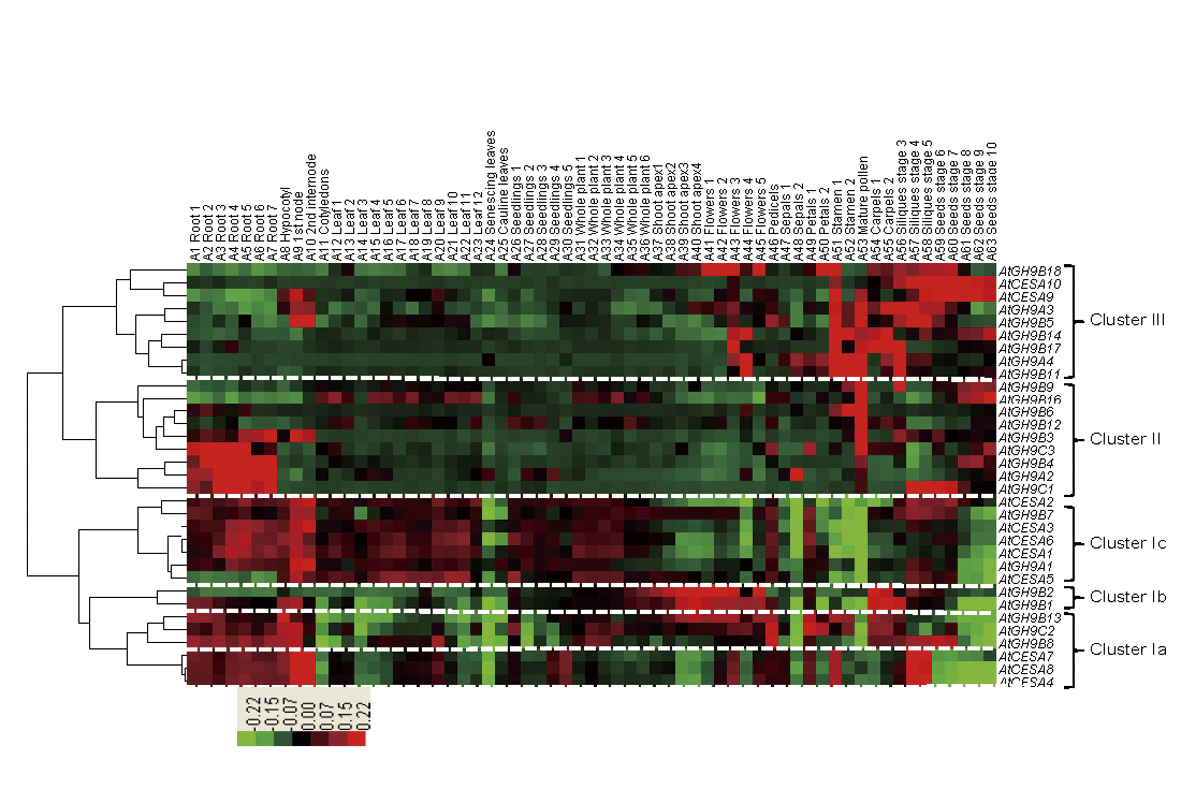

Supplement: Figure S3 — Co-expression profiling among AtGH9 and AtCESA family genes in Arabidopsis. The expression profiling of AtGH9 and AtCESA family genes based on Arabidopsis microarray data GSE5629, GSE5630, GSE5631, GSE5632, GSE5633 and GSE5634, and performed by the hierarchical cluster analysis. (TIF) [file pone.0050171.s003.tif]

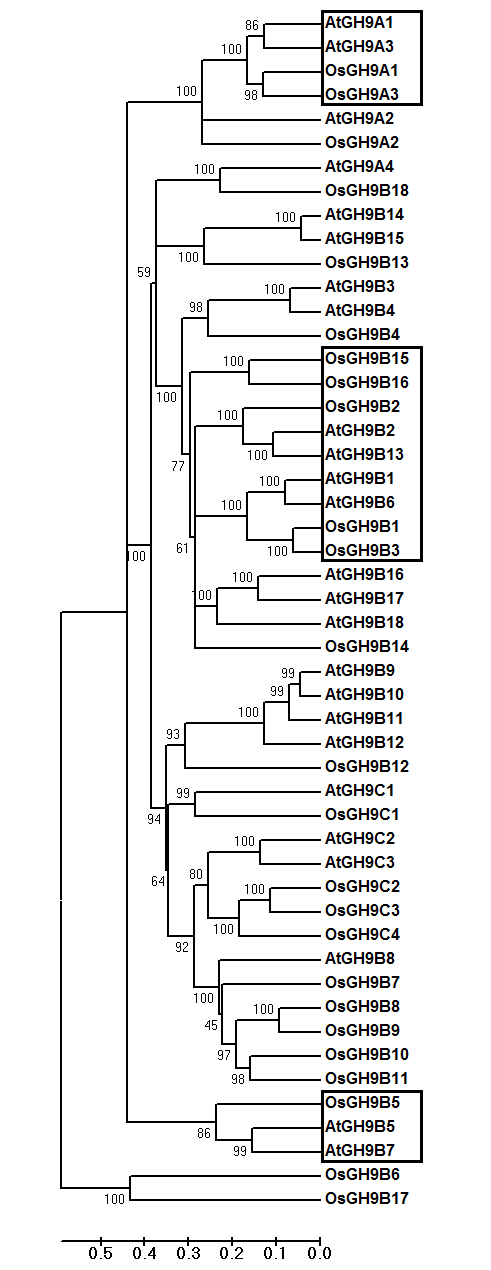

Supplement: Figure S4 — Phylogenetic comparison of GH9 families in rice and Arabidopsis. The sequences of GH9 family proteins obtained from rice (http://rice.plantbiology.msu.edu/) and Arabidopsis (http://www.arabidopsis.org/) were aligned with Clustal X program and then constructed a phylogenetic tree using MEGA3.1 software. (TIF) [file pone.0050171.s004.tif]
