# Supplementary material for: Global Identification of Multiple OsGH9 Family Members and Their Involvement in Cellulose Crystallinity Modification in Rice
Source: PLoS One. 2013 Jan 4;8(1):e50171. doi: 10.1371/journal.pone.0050171 (PMC3537678; doi:10.1371/journal.pone.0050171)
Supplement: Table S1 — Information of 25 OsGH9 genes in rice. (DOCX) [file pone.0050171.s005.docx]

**Table S1 Information of 25 *OsGH9* genes in rice.**

| No. | Gene name | TIGR  loci③ | KOME  cDNA④ | No of amino acids⑤ | pI  ⑥ | Membrane anchored helix amino acids |
| --- | --- | --- | --- | --- | --- | --- |
| 1 | *OsGH9A1* | LOC_Os03g21210 | AK102748 | 619 | 9.24 | 74-96 |
| 2 | *OsGH9A2* | LOC_Os04g41970 | AK243593 | 641 | 9.15 | 73-95 |
| 3 | *OsGH9A3* | LOC_Os03g52630 | AK070408 | 620 | 9.06 | 72-94 |
| 4 | *OsGH9B1* | LOC_Os02g50040 | AK101108 | 508 | 7.67 | 12-34 |
| 5 | *OsGH9B2* | LOC_Os01g21070 | AK106887 | 499 | 8.75 | None② |
| 6 | *OsGH9B3* | LOC_Os06g14540 | AK060686 | 497 | 6.57 | None |
| 7 | *OsGH9B4* | LOC_Os04g36610 | NF① | 500 | 8.15 | 13-35 |
| 8 | *OsGH9B5* | LOC_Os09g36350 | AK100449 | 528 | 6.00 | 7-29 |
| 9 | *OsGH9B6* | LOC_Os06g50140 | AK121369 | 518 | 5.56 | 7-29 |
| 10 | *OsGH9B7* | LOC_Os02g05744 | AK120536 | 534 | 5.27 | 5-29 |
| 11 | *OsGH9B8* | LOC_Os02g50490 | AK099698 | 553 | 6.29 | 27-49 |
| 12 | *OsGH9B9* | LOC_Os06g13830 | AK065325 | 538 | 5.89 | None |
| 13 | *OsGH9B10* | LOC_Os08g32940 | NF | 525 | 6.97 | None |
| 14 | *OsGH9B11* | LOC_Os09g23084 | AK105580 | 441 | 6.18 | none |
| 15 | *OsGH9B12* | LOC_Os05g03840 | NF | 512 | 5.20 | None |
| 16 | *OsGH9B13* | LOC_Os08g29770 | AK071784 | 516 | 5.51 | None |
| 17 | *OsGH9B14* | LOC_Os09g36060 | AK106815 | 515 | 5.40 | 17-39 |
| 18 | *OsGH9B15* | LOC_Os02g03120 | AK106726 | 503 | 5.55 | None |
| 19 | *OsGH9B16* | LOC_Os08g02220 | AK106851 | 523 | 8.44 | None |
| 20 | *OsGH9B17* | LOC_Os12g24040 | NF | 694 | 8.98 | 669-691 |
| 21 | *OsGH9B18* | LOC_Os02g53820 | AK100098 | 501 | 8.02 | 13-35 |
| 22 | *OsGH9C1* | LOC_Os04g57860 | AK119913 | 625 | 5.56 | 13-35 |
| 23 | *OsGH9C2* | LOC_Os01g12030 | NF | 640 | 9.36 | 7-29 |
| 24 | *OsGH9C3* | LOC_Os01g12070 | AK103304 | 640 | 6.52 | 7-29 |
| 25 | *OsGH9C4* | LOC_Os05g12150 | AK099370 | 629 | 8.30 | 7-29 |

**Note:** ① NF: not found. ② None: there was not membrane-anchored helix. ③TIGR loci: <http://rice.plantbiology.msu.edu/>. ④KOME cDNA: <http://cdna01>.dna.affrc.go.jp/cDNA /. ⑤No of AA: number of total amino acid residues in GH9 protein. ⑥pI: isoelectric point of a GH9 protein
