# Supplementary material for: Global Identification of Multiple OsGH9 Family Members and Their Involvement in Cellulose Crystallinity Modification in Rice
Source: PLoS One. 2013 Jan 4;8(1):e50171. doi: 10.1371/journal.pone.0050171 (PMC3537678; doi:10.1371/journal.pone.0050171)
Supplement: Table S2 — Correlation coefficients between OsGH9 and OsCESA expression levels in 66 tissues of ZS97 and MH63 (n = 66). (DOCX) [file pone.0050171.s006.docx]

**Table S2 Correlation coefficients between *OsGH9* and *OsCESA* expression levels in 66 tissues of *ZS97* and *MH63* (n=66).**

| Pairs | *CESA1* | *CESA2* | *CESA3* | *CESA4* | *CESA5* | *CESA6* | *CESA7* | *CESA8* | *CESA9* | *CESA11* |
| --- | --- | --- | --- | --- | --- | --- | --- | --- | --- | --- |
| *GH9A3* | **.866**** | .352** | **.880**** | **.825**** | .535** | .595** | .773** | **.875**** | .767** | 0.124 |
| *GH9B5* | **.891**** | .271* | .739** | .763** | .618** | .769** | **.800**** | **.859**** | .795** | -0.079 |
| *GH9B1* | .388** | .558** | 0.215 | 0.199 | .419** | .601** | 0.241 | 0.228 | 0.205 | -0.025 |
| *GH9B2* | .254* | .260* | -0.028 | 0 | .384** | .521** | 0.108 | 0.127 | 0.079 | -0.218 |
| *GH9B3* | .260* | .375** | 0.004 | 0.133 | .337** | .483** | 0.165 | 0.175 | 0.174 | -0.208 |
| *GH9B16* | .323** | .342** | 0.026 | 0.07 | .488** | .596** | 0.127 | 0.176 | 0.12 | -.246* |
| *GH9B8* | .537** | -0.088 | .524** | .710** | .286* | .324** | .719** | .650** | .696** | 0.067 |
| *GH9B9* | .751** | 0.001 | .651** | .649** | .523** | .585** | .753** | .789** | .703** | 0.078 |
| *GH9B10* | .547** | 0.226 | .493** | .750** | .471** | .347** | .668** | .615** | .651** | .319** |
| *GH9B11* | .354** | -0.037 | .350** | .633** | .322** | 0.123 | .573** | .505** | .548** | .262* |
| *GH9A1* | -0.049 | .433** | -0.203 | -0.146 | .311* | 0.15 | -0.227 | -0.152 | -0.197 | -0.097 |
| *GH9A2* | .599** | -0.039 | .361** | .409** | .603** | .495** | .445** | .655** | .457** | -0.086 |
| *GH9B18* | -0.079 | 0.103 | 0.001 | -0.144 | -0.04 | 0.028 | -0.2 | -0.123 | -0.211 | 0.201 |
| *GH9B4* | 0.124 | .336** | 0.094 | 0.036 | .301* | .273* | -0.051 | 0.065 | -0.034 | .286* |
| *GH9B6* | -0.064 | -0.001 | 0.126 | 0.196 | -0.221 | -0.126 | 0.099 | 0.03 | 0.157 | .288* |
| *GH9B12* | -0.169 | 0.216 | -0.037 | 0.222 | -0.179 | -.269* | -0.024 | -0.099 | 0.052 | .277* |
| *GH9B13* | -0.13 | -.332** | 0.124 | 0.119 | -.496** | -.357** | 0.168 | 0 | 0.109 | .254* |
| *GH9B14* | .308* | .348** | .265* | .309* | .332** | .404** | .253* | .270* | .317** | 0.007 |
| *GH9B15* | 0.148 | -0.08 | 0.011 | -0.091 | .278* | .302* | 0.011 | 0.112 | -0.011 | -0.026 |
| *GH9B17* | -.250* | 0.142 | -0.117 | -0.154 | -0.18 | -0.102 | -.283* | -.275* | -0.201 | 0.105 |
| *GH9C1* | 0.041 | -.442** | 0.05 | 0.106 | -0.057 | -0.082 | 0.225 | 0.113 | 0.194 | 0.107 |
| *GH9C2* | -.245* | -0.015 | -0.234 | -.253* | -0.23 | -0.184 | -.311* | -.287* | -.270* | -0.173 |
| *GH9C3* | .392** | 0.02 | 0.162 | .280* | .346** | .318** | .389** | .396** | .337** | -0.122 |
| *GH9C4* | -0.024 | -0.044 | -0.218 | -0.128 | 0.177 | 0.155 | -0.139 | -0.078 | -0.122 | -0.122 |

* and **: signiﬁcance test at *p* < 0.05 and 0.01, respectively; Total of 66 data (n=66) were from the cDNA chip data of 33 tissues of two rice varieties (*ZS97* and *MH63)* each at CREP database <http://crep.ncpgr.cn> as shown in Figure 2. The bold data indicated the relatively high correlation coefficient values.
