# Supplementary material for: Global Identification of Multiple OsGH9 Family Members and Their Involvement in Cellulose Crystallinity Modification in Rice
Source: PLoS One. 2013 Jan 4;8(1):e50171. doi: 10.1371/journal.pone.0050171 (PMC3537678; doi:10.1371/journal.pone.0050171)
Supplement: Table S3 — Correlation coefficients among OsGH9 expression levels in 66 tissues of ZS97 and MH63 (n = 66). (DOCX) [file pone.0050171.s007.docx]

**Table S3 Correlation coefficients among *OsGH9* expression levels in 66 tissues of *ZS97* and *MH63* (n=66).**

| Pairs | *GH9A3* | *GH9B5* | *GH9B8* | *GH9B9* | *GH9B10* | *GH9B11* | *GH9B1* | *GH9B2* | *GH9B3* | *GH9B16* | *GH9A2* | *GH9A1* | *GH9B18* | *GH9B4* | *GH9B6* | *GH9B12* | *GH9B13* | *GH9B14* | *GH9B15* | *GH9B17* | *GH9C1* | *GH9C2* | *GH9C3* | *GH9C4* |
| --- | --- | --- | --- | --- | --- | --- | --- | --- | --- | --- | --- | --- | --- | --- | --- | --- | --- | --- | --- | --- | --- | --- | --- | --- |
| *GH9A3* | 1 | .821** | .578** | .622** | .596** | .450** | 0.224 | -0.01 | 0.086 | 0.079 | .386** | -0.1 | -0.071 | 0.08 | 0.16 | 0.105 | 0.068 | .258* | -0.096 | -0.172 | 0.072 | -.258* | .262* | -.257* |
| *GH9B5* |  | 1 | .492** | .656** | .453** | .314* | .511** | .365** | .445** | .434** | .458** | -0.101 | -0.107 | 0.011 | 0.006 | -0.165 | -0.156 | .469** | 0.185 | -.344** | 0.051 | -0.211 | .418** | -0.025 |
| *GH9B8* |  |  | 1 | .738** | .793** | .672** | -0.184 | -.268* | -.251* | -0.231 | .481** | -.392** | -0.184 | -0.024 | 0.153 | 0.165 | .373** | -0.002 | -0.174 | -0.044 | .251* | -.372** | 0.14 | -0.183 |
| *GH9B9* |  |  |  | 1 | .684** | .550** | 0.06 | 0.04 | -0.045 | 0.088 | .704** | -.277* | -0.162 | 0.113 | -0.086 | -0.094 | 0.114 | 0.07 | 0.097 | -0.163 | .307* | -.303* | .339** | -0.004 |
| *GH9B10* |  |  |  |  | 1 | .767** | -0.039 | -0.199 | -0.15 | -0.123 | .487** | -0.2 | -0.099 | 0.181 | 0.175 | .295* | 0.208 | 0.101 | -0.163 | -0.076 | 0.15 | -.347** | 0.141 | -0.128 |
| *GH9B11* |  |  |  |  |  | 1 | -0.168 | -.248* | -0.133 | -0.196 | .459** | -.263* | -0.198 | -0.047 | 0.204 | 0.18 | 0.223 | 0.071 | -0.071 | -0.147 | 0.172 | -0.235 | 0.205 | -0.025 |
| *GH9B1* |  |  |  |  |  |  | 1 | .790** | **.806**** | .761** | -0.006 | .321** | 0.127 | 0.076 | -0.164 | -.277* | -.502** | .625** | .419** | -0.121 | -.305* | 0.031 | .314* | .256* |
| *GH9B2* |  |  |  |  |  |  |  | 1 | **.879**** | **.916**** | 0.161 | .391** | 0.035 | 0.197 | -.268* | -.419** | -.673** | .408** | .665** | -0.17 | -0.045 | -0.037 | .563** | .536** |
| *GH9B3* |  |  |  |  |  |  |  |  | 1 | **.855**** | 0.045 | .295* | 0.098 | 0.116 | -0.033 | -.298* | -.602** | .602** | .555** | -0.226 | -0.195 | 0.041 | .473** | .424** |
| *GH9B16* |  |  |  |  |  |  |  |  |  | 1 | 0.238 | .365** | 0.032 | .299* | -0.232 | -.322** | -.700** | .427** | .631** | -0.077 | -0.016 | 0.04 | .590** | .593** |
| *GH9A2* |  |  |  |  |  |  |  |  |  |  | 1 | -0.06 | -0.158 | 0.235 | -0.217 | -0.186 | -0.169 | -0.015 | .338** | -0.219 | .409** | -0.231 | .523** | .304* |
| *GH9A1* |  |  |  |  |  |  |  |  |  |  |  | 1 | -0.088 | 0.126 | -.352** | 0.07 | -.669** | 0.114 | 0.095 | 0.065 | -.348** | 0.009 | 0.113 | 0.183 |
| *GH9B18* |  |  |  |  |  |  |  |  |  |  |  |  | 1 | .334** | .429** | 0.116 | 0.049 | 0.168 | 0.198 | 0.13 | 0.096 | 0.199 | -0.072 | 0.037 |
| *GH9B4* |  |  |  |  |  |  |  |  |  |  |  |  |  | 1 | 0.232 | 0.173 | -0.22 | 0.081 | .321** | 0.198 | .271* | -0.052 | 0.173 | .312* |
| *GH9B6* |  |  |  |  |  |  |  |  |  |  |  |  |  |  | 1 | .314* | .427** | 0.162 | -0.021 | 0.111 | .250* | 0.14 | -0.062 | -0.144 |
| *GH9B12* |  |  |  |  |  |  |  |  |  |  |  |  |  |  |  | 1 | 0.163 | -0.128 | -.458** | .325** | 0.001 | -0.146 | -0.176 | -.253* |
| *GH9B13* |  |  |  |  |  |  |  |  |  |  |  |  |  |  |  |  | 1 | -.308* | -.408** | 0.083 | 0.143 | 0.007 | -.383** | -.465** |
| *GH9B14* |  |  |  |  |  |  |  |  |  |  |  |  |  |  |  |  |  | 1 | .408** | -0.077 | -.307* | 0.109 | 0.149 | 0.131 |
| *GH9B15* |  |  |  |  |  |  |  |  |  |  |  |  |  |  |  |  |  |  | 1 | -0.098 | .256* | 0.112 | .587** | .628** |
| *GH9B17* |  |  |  |  |  |  |  |  |  |  |  |  |  |  |  |  |  |  |  | 1 | -0.043 | 0.197 | -.279* | 0.166 |
| *GH9C1* |  |  |  |  |  |  |  |  |  |  |  |  |  |  |  |  |  |  |  |  | 1 | -0.23 | .470** | 0.203 |
| *GH9C2* |  |  |  |  |  |  |  |  |  |  |  |  |  |  |  |  |  |  |  |  |  | 1 | -0.142 | 0.206 |
| *GH9C3* |  |  |  |  |  |  |  |  |  |  |  |  |  |  |  |  |  |  |  |  |  |  | 1 | .487** |
| *GH9C4* |  |  |  |  |  |  |  |  |  |  |  |  |  |  |  |  |  |  |  |  |  |  |  | 1 |

* and **: signiﬁcance test at *p* < 0.05 and 0.01, respectively. Total of 66 data (n=66) were from the cDNA chip data of 33 tissues of two rice varieties (*ZS97* and *MH63)* each at CREP database http://crep. ncpgr.cn as shown in Figure 2. The bold data indicated the relatively high correlation coefficient values.
