# Supplementary material for: Global Identification of Multiple OsGH9 Family Members and Their Involvement in Cellulose Crystallinity Modification in Rice
Source: PLoS One. 2013 Jan 4;8(1):e50171. doi: 10.1371/journal.pone.0050171 (PMC3537678; doi:10.1371/journal.pone.0050171)
Supplement: Table S4 — Transcript levels (%) changes of OsGH9 and OsCESA in four internodes of mutants (fc4 and fc11) and wild type (NPB) at booting stages. (DOCX) [file pone.0050171.s008.docx]

**Table S4 Transcript levels (%) changes of *OsGH9* and *OsCESA* in four internodes of mutants (*fc4* and *fc11*) and wild type (*NPB*) at booting stages.**

|  | NPB internodes | | | |  | *fc4* internodes | | | |  | *fc11* internodes | | | |
| --- | --- | --- | --- | --- | --- | --- | --- | --- | --- | --- | --- | --- | --- | --- |
| Genes | 1st | 2nd | 3rd | 4th |  | 1st | 2nd | 3rd | 4th |  | 1st | 2nd | 3rd | 4th |
| **Cluster Ia** |  |  |  |  |  |  |  |  |  |  |  |  |  |  |
| *CESA4* | 0.56 | 3.30 | 14.20 | 22.80 |  | 1.02 | 1.68 | 8.31 | 9.57 |  | 0.69 | 6.38 | 0.10 | 0.06 |
| *CESA7* | 0.20 | 1.36 | 5.54 | 9.05 |  | 0.32 | 4.33 | 4.53 | 0.06 |  | 0.13 | 1.28 | 1.81 | 3.82 |
| *CESA9* | 1.98 | 14.03 | 14.46 | 16.03 |  | 0.38 | 0.04 | 0.02 | 0.23 |  | 3.88 | 13.79 | 5.72 | 7.66 |
| *GH9A1* | 1.52 | 21.74 | 5.42 | 5.73 |  | 5.79 | 24.00 | 67.84 | 3.11 |  | 1.76 | 9.28 | 1.83 | 3.46 |
| *GH9B8* | 2.88 | 1.47 | 1.37 | 2.78 |  | 1.41 | 6.16 | 3.24 | 4.71 |  | 2.28 | 7.00 | 3.93 | 1.82 |
| *GH9B9* | 0.62 | 0.41 | 0.90 | 0.10 |  | 0.24 | 44.38 | 12.50 | 0.42 |  | 0.71 | 0.73 | 0.03 | 0.06 |
| *GH9B11* | 1.82 | 1.10 | 0.34 | 0.40 |  | 0.26 | 0.73 | 2.49 | 3.62 |  | 0.07 | 0.28 | 0.14 | 0.42 |
| **Cluster Ib**  *CESA1* | 18.48 | 19.60 | 56.41 | 4.59 |  | 3.76 | 13.68 | 0.25 | 0.28 |  | 5.64 | 6.58 | 8.75 | 0.27 |
| *CESA3* | 12.39 | 30.77 | 50.14 | 43.46 |  | 8.04 | 29.16 | 5.20 | 6.00 |  | 16.85 | 33.21 | 43.04 | 0.14 |
| *CESA8* | 11.41 | 22.99 | 38.00 | 15.14 |  | 21.27 | 46.94 | 5.83 | 2.58 |  | 10.97 | 12.42 | 16.07 | 1.79 |
| *GH9A3* | 47.99 | 67.58 | 81.66 | 55.90 |  | 55.55 | 117.94 | 9.20 | 16.67 |  | 36.93 | 30.05 | 61.05 | 3.10 |
| *GH9B5* | 10.31 | 12.59 | 14.40 | 8.14 |  | 11.78 | 23.11 | 2.34 | 1.59 |  | 7.66 | 10.96 | 8.59 | 0.81 |
| **Cluster IIa** |  |  |  |  |  |  |  |  |  |  |  |  |  |  |
| *GH9B1* | 7.11 | 7.66 | 0.14 | 0.08 |  | 10.08 | 0.71 | 0.23 | 0.24 |  | 8.71 | 0.46 | 0.05 | 0.22 |
| *GH9B3* | 2.41 | 1.14 | 0.03 | 0.03 |  | 0.52 | 0.03 | 0.01 | 0.07 |  | 1.45 | 0.01 | 0.01 | 0.11 |
| *GH9B16* | 5.07 | 1.73 | 0.05 | 0.01 |  | 3.42 | 0.17 | 0.09 | 0.05 |  | 2.79 | 0.15 | 0.36 | 0.03 |

**Note:** Clusters Ia, Ib and IIa in microarray chip were selected in the expression pattern analysis in the rice internodes. The transcript levels of these genes were detected as the percentage of their expressions relative to internal standard gene *Ubiquitin*.
