# Supplementary material for: Global Identification of Multiple OsGH9 Family Members and Their Involvement in Cellulose Crystallinity Modification in Rice
Source: PLoS One. 2013 Jan 4;8(1):e50171. doi: 10.1371/journal.pone.0050171 (PMC3537678; doi:10.1371/journal.pone.0050171)
Supplement: Table S5 — Correlation coefficients between OsGH9 genes expression level and cellulase specific activity or lignocellulose CrI in 12 internodes of mutants (fc4 and fc11) and wild type (NPB) at booting stages (n = 12). (DOCX) [file pone.0050171.s009.docx]

**Table S5 Correlation coefficients between *OsGH9* genes expression level and cellulase specific activity or lignocellulose CrI in 12 internodes of mutants (*fc4* and *fc11*) and wild type (*NPB*) at booting stages (n=12).**

| Pairs | Cellulase specific activity | CrI of Lignocellulose(%) |
| --- | --- | --- |
| **Cluster Ia**  GH9B8  GH9B9  GH9B11  **Cluster Ib**  GH9A3 | 0.060  0.230  0.190  0.360 | 0.180  -0.160  -0.100  0.120 |
| GH9B5 | 0.570 | -0.090 |
| **Cluster IIa**  GH9A1 | 0.200 | 0.290 |
| GH9B1  GH9B3 | **0.818****  0.530 | **-0.902****  **-0.716**** |
| GH9B16 | **0.855**** | **-0.813**** |

**: signiﬁcance test at *p* < 0.01. Total of 12 data (n=12) were from four internodes of two mutants and one wild type (3x4) as shown in Figure 4 and Table S4. The bold data indicated the relatively high correlation coefficient values.
