# Supplementary material for: Global Identification of Multiple OsGH9 Family Members and Their Involvement in Cellulose Crystallinity Modification in Rice
Source: PLoS One. 2013 Jan 4;8(1):e50171. doi: 10.1371/journal.pone.0050171 (PMC3537678; doi:10.1371/journal.pone.0050171)
Supplement: Table S6 — Correlation coefficients between OsGH9 and OsCESA expression levels in rice at booting stage (n = 12). (DOCX) [file pone.0050171.s010.docx]

**Table S6 Correlation coefficients between *OsGH9* and *OsCESA* expression levels in rice at booting stage (n=12).**

| Pairs | CESA1 | CESA3 | CESA8 | CESA4 | CESA7 | CESA9 |
| --- | --- | --- | --- | --- | --- | --- |
| **Cluster Ia** |  |  |  |  |  |  |
| GH9A1 | 0.399 | **0.636*** | **0.601*** | **0.587*** | 0.476 | 0.455 |
| GH9B8 | 0.182 | 0.343 | -0.098 | -0.385 | -0.035 | 0.531 |
| GH9B9 | 0.203 | 0.014 | 0.210 | 0.378 | 0.098 | -0.385 |
| GH9B11 | 0.522 | 0.140 | 0.340 | -0.095 | 0.161 | 0.319 |
| **Cluster Ib** |  |  |  |  |  |  |
| GH9A3 | **0.797**** | **0.734**** | **0.951**** | 0.168 | 0.336 | 0.280 |
| GH9B5 | **0.790**** | **0.587*** | **0.944**** | 0.154 | 0.203 | 0.168 |
| **Cluster IIa** |  |  |  |  |  |  |
| GH9B1 | 0.091 | -0.343 | 0.098 | -0.238 | **-0.636*** | -0.350 |
| GH9B3 | 0.152 | -0.332 | -0.116 | -0.377 | -0.526 | -0.042 |
| GH9B16 | 0.361 | -0.088 | 0.249 | -0.522 | **-0.588*** | -0.329 |

* and ** signiﬁcance test at *p* < 0.05 and 0.01, respectively. Total of 12 data (n=12) were from four internodes of two mutants and one wild type (3x4) as shown in Figure 4 and Table S4. The bold data indicated the relatively high correlation coefficient values.
