# Supplementary material for: Global Identification of Multiple OsGH9 Family Members and Their Involvement in Cellulose Crystallinity Modification in Rice
Source: PLoS One. 2013 Jan 4;8(1):e50171. doi: 10.1371/journal.pone.0050171 (PMC3537678; doi:10.1371/journal.pone.0050171)
Supplement: Table S7 — Primer pairs of genes for qRT-PCR. (DOCX) [file pone.0050171.s011.docx]

**Table S7 Primer pairs of genes for qRT-PCR.**

| Gene | Forward primers | Reverse primers |
| --- | --- | --- |
| GH9A1 | AACCCTAACATCATCGTCGGA | CGGCACGGCAGAAAACAT |
| GH9A2 | TACACCGTCGCCAACTCCT | TCGGGTTATCCCCAAGAATG |
| GH9A3 | CTGGTGGCAGCCTTGATTT | TGTCGTTCCCTCCGTTTGA |
| GH9B1 | ACTTCCTCTACAAAACCCCAAC | ATAATCCACTCGCTGAACCATA |
| GH9B2 | CCACGGACCAGACGCAGT | GTGAGCAGCAGGAAGGAGC |
| GH9B3 | TCCGATCTCCTCTACGACTGA | CCCCGTTCCTGCCATTT |
| GH9B5 | GAGCCCAAACCCAAACG | ACCAGAAAGAAGACCAACGAG |
| GH9B6 | GCCACCACCTACAACAGCG | CCGTCAGAATCCGAGCAAT |
| GH9B8 | TTCCCCATTCTTTTGTTCATTT | CCACCCGTCCCTTTCTCTT |
| GH9B9 | GGGATTTGCATTTGCTTGC | GCCCCTATTCTTTTGTTCATTC |
| GH9B11 | CCGCCCAAATTAAAACCAC | GTCTTCGCCAGGCTACACC |
| GH9B15 | ACGGCTTCCAGTCCCAGTA | GCCCCGAGTATGTAGTCCA |
| GH9B16 | CGGAACCACCAAGTAGCCAT | GAATACACGACAAAGTCAACGAAC |
| GH9B18 | ACCAGGTCAACTGCGGGAT | ACTGGGTGGCGATGAGGTT |
| GH9C1 | TCACCATCGTCTACATCCAGG | AAGCAGCAGCCAACCACAC |
| GH9C2 | GTGTTGCAGGAGTTGGTTTGCGTA | TTCTGCAGGTTGGAGATGGTGACT |
| GH9C3 | AGGTGTATGGCTAGTGTTTGGA | CTGCTCTACTGCCTGGATGAT |
| GH9C4 | GTGGTAGAGAGAGAGTAGAGTGTGG | AGGGCAAAAGGAGGACGAT |
| CESA1 | GGCATGGTGGCGGGTATAT | AGGCGACGGCTTTCTGAGT |
| CESA3 | GTGTAGATAGAAACACGAAAGGTG | CAATGGGCACCCACACACGT |
| CESA4 | CCATCGTCTACCCGTTCACC | TCCAGCCCAGCAATCATCTT |
| CESA7 | TTAACGGGGTTCAAGATGC | ATGGTGTTGGTGTAGGCGA |
| CESA8 | TGGTCGGTGTTGTTGCTGG | ATGGTCGGAGTGCGGTTTT |
| CESA9 | TACAAGAACGGCAACCTCA | AAGAACAAACTCGCAAACG |
| UBQ | CCAGGACAAGATGATCTGCC | AAGAAGCTGAAGCATCCAGC |
