# Supplementary material for: Global Identification of Multiple OsGH9 Family Members and Their Involvement in Cellulose Crystallinity Modification in Rice
Source: PLoS One. 2013 Jan 4;8(1):e50171. doi: 10.1371/journal.pone.0050171 (PMC3537678; doi:10.1371/journal.pone.0050171)
Supplement: Table S8 — List of 25 putative motifs in the OsGH9 family proteins. (DOCX) [file pone.0050171.s012.docx]

| **Motif** | **Width** | **Amino acid sequences** |
| --- | --- | --- |
| 1 | 41 | [FL]A[RK]SQ[VA]DY[IV]LG[DSK]NP[LMR][GK]MSY[ML]VG[YF]GA[RK][YF]PRR[VI]HHR[GA][AS]S[IL][PV]S[IV] |
| 2 | 29 | VDL[VT]GGYYDAGD[NH]VKFG[FL]PMAF[TS][VAM]TM[LM][SA]W |
| 3 | 29 | [YW][VA][QE]VGD[GP]D[ST]DHYCW[EQ]RPEDM[DT]T[PS]R[TQP][AV]Y[KR][VI] |
| 4 | 29 | A[VIL]VGGPDR[NR]DAFAD[ED]R[DG]N[YF]MQ[TS]E[PA][TAC]TY[NI][NT] |
| 5 | 21 | [YF][CYA][SN][SYF][ST]GYQDELLW[AG]A[ALS]WL[HY]RA |
| 6 | 23 | [HF]DY[AG]DALAK[SA][IL]L[FY]FE[AG]QRSG[RK]LP |
| 7 | 29 | GG[LM]L[YF][VHK]R[GR]W[NS]N[LM]Q[YH]V[TV][SN]A[AS]FL[LA][ALT][VA]Y[SA][DK]Y[LM] |
| 8 | 21 | PGSD[VL]A[AG]ETAAA[LM]AAAS[IL]VF[RK] |
| 9 | 29 | A[AG]GEL[GD][HN][AV][RL][DE]A[IV][RK]WG[TA]DY[LF][LV]KAH[TAP][SA]P[ND]VL |
| 10 | 29 | PAY[SA]x[RL]LLHH[AS][KQ]Q[LV]F[ED]FAD[KT][YH]RG[KS]Y[DS][DS]S[IL] |
| 11 | 21 | W[AS][IV][NT]EF[SG]WD[NVD]K[YL][AP]G[AV]Q[VI]L[LA][AS][KR] |
| 12 | 29 | H[ADR][EG]VL[QE]x[YF][RK]x[KN]A[DE]N[FY][IMV]C[SA][LC][LV][PG][GKR][SN][AP][SG][FG][GNQ]AT |
| 13 | 15 | N[QN]R[VI][AR]WR[GA][DN]S[GA]L[TS]DG |
| 14 | 15 | [KS][WY][LFY]G[SRT]GG[PA]NPN[VL]LVG |
| 15 | 15 | [TS][GR][NRD][RA][ST]YL[DS]Y[AIL][TV]DN[AG]D |
| 16 | 11 | [HDS]PA[FR][IV][GS]CK[EQ]G[FY] |
| 17 | 11 | AP[LM]VGV[LF]A[RY][LF]A |
| 18 | 50 | KK[KD]KYVDLGC[LMV]V[VL][DKS]RK[LI][FL][MVW]W[TV][VL][GW][TV][LI]L[AG][AV][AFG][ILV][FL][IA]G[LF][VP][AMV][GIM]I[AV]K[ALS][IV]P[HKR][KH][HKR][PR][PH][PA]P |
| 19 | 8 | SV[LIV]E[YF]G[DAK]x |
| 20 | 21 | [VI]DKNT[MI]FSAVPPMFP[AST][PT]PPPP |
| 21 | 21 | [SA]SSxAT[VL]SC[GS][GA]GAV[ST]PA[DT][LI][RL][KS] |
| 22 | 28 | [EGI][IFT][EQV][QHL][NKT][AV]T[AGN][ST]W[KAE][KAR][DNR][GR][RV][TD]Y[YHR]R[YH][AV][VA]T[VA][SKT]N[RT][SC] |
| 23 | 22 | GKTV[ER]ELH[IL]G[IV][GS][KG][LP][HRY]G[PR][VL]WGLE |
| 24 | 10 | [RH][QT]LDETQQSW |
| 25 | 13 | M[YF][GS][RA][DN][PH]WGG[PS][LF]EI |

**Table S8 List of 25 putative motifs in the OsGH9 family proteins.**
