# Supplementary material for: Global Identification of Multiple OsGH9 Family Members and Their Involvement in Cellulose Crystallinity Modification in Rice
Source: PLoS One. 2013 Jan 4;8(1):e50171. doi: 10.1371/journal.pone.0050171 (PMC3537678; doi:10.1371/journal.pone.0050171)
Supplement: Table S9 — Tissue samples from 33 developmental stages of two rice varieties. (DOCX) [file pone.0050171.s013.docx]

**Table S9 Tissue samples from 33 developmental stages of two rice varieties.**

| **Sample No** | **Abbreviation** | **Tissues or organ** | **Developmental stages** |
| --- | --- | --- | --- |
| M1(Minghui)/Z1(Zhenshan) | Calli 1 | Calli | 15 DAS (days after subculture) |
| M2(Minghui)/Z2(Zhenshan) | Calli 2 | Calli | 5 DAR (days after regeneration) |
| M3(Minghui)/Z3(Zhenshan) | Calli 3 | Calli | Screening stage |
| M4(Minghui)/Z4(Zhenshan) | Calli 4 | Calli | 15 DAI T2 (days after induction) |
| M5(Minghui)/Z5(Zhenshan) | Calli 5 | Calli | 15 DAI T3 (days after induction) |
| M6(Minghui)/Z6(Zhenshan) | Seed imbibition | Seed | 72 h after imbibition |
| M7(Minghui)/Z7(Zhenshan) | Seed germination | Embryo bud and radicle | 3 days after germination |
| M8(Minghui)/Z8(Zhenshan) | Plumule/Dark | Plumule | 48 hours after emergence, Dark |
| M9(Minghui)/Z9(Zhenshan) | Plumule/Light | Plumule | 48 hours after emergence, Light |
| M10(Minghui)/Z10(Zhenshan) | Radicle/Dark | Radicle | 48 hours after emergence, Dark |
| M11(Minghui)/Z11(Zhenshan) | Radicle/Light | Radicle | 48 hours after emergence, Light |
| M12(Minghui)/Z12(Zhenshan) | Seedlings | Seedling | Three-leaf stage |
| M13(Minghui)/Z13(Zhenshan) | Young shoot | Shoot | Seedlings with 2 tillers |
| M14(Minghui)/Z14(Zhenshan) | Young root | Root | Seedlings with 2 tillers |
| M15(Minghui)/Z15(Zhenshan) | Mature leaf | Leaf | Stage 3(secondary branch primordium differentiation stage) |
| M16(Minghui)/Z16(Zhenshan) | Old leaf | Leaf | 4–5 cm young panicle |
| M17(Minghui)/Z17(Zhenshan) | Mature sheath | Sheath | Stage 3 |
| M18(Minghui)/Z18(Zhenshan) | Old sheath | Sheath | 4–5 cm young panicle |
| M19(Minghui)/Z19(Zhenshan) | Young flag leaf | Flag leaf | 5 DBH (5 days before heading) |
| M20(Minghui)/Z20(Zhenshan) | Old flag leaf | Flag leaf | 14 DAH (14 days after heading) |
| M21(Minghui)/Z21(Zhenshan) | Panicle, stage 3 | Young Panicle | Stage 3 |
| M22(Minghui)/Z22(Zhenshan) | Panicle, stage 4 | Young Panicle | Stage 4 (pistil/stamen primordium differentiation stage) |
| M23(Minghui)/Z23(Zhenshan) | Panicle, stage 5 | Young Panicle | Stage 5 (pollen-mother cell formation stage) |
| M24(Minghui)/Z24(Zhenshan) | Young panicle | Panicle | 4–5 cm young panicle |
| M25(Minghui)/Z25(Zhenshan) | Old panicle | Panicle | Heading stage |
| M26(Minghui)/Z26(Zhenshan) | Young stem | Stem | 5 DBH |
| M27(Minghui)/Z27(Zhenshan) | Old stem | Stem | Heading stage |
| M28(Minghui)/Z28(Zhenshan) | Hull | Hull | 1 DBF (1 day before flowering) |
| M29(Minghui)/Z29(Zhenshan) | Spikelet | Spikelet | 3 DAP (3 days after pollination) |
| M30(Minghui)/Z30(Zhenshan) | Stamen | Stamen | 1 DBF |
| M31(Minghui)/Z31(Zhenshan) | Endosperm 1 | Endosperm | 7 DAP (7 days after pollination) |
| M32(Minghui)/Z32(Zhenshan) | Endosperm 2 | Endosperm | 14 DAP (14 days after pollination) |
| M33(Minghui)/Z33(Zhenshan) | Endosperm 3 | Endosperm | 21 DAP (21 days after pollination) |
