# Supplementary material for: Global Identification of Multiple OsGH9 Family Members and Their Involvement in Cellulose Crystallinity Modification in Rice
Source: PLoS One. 2013 Jan 4;8(1):e50171. doi: 10.1371/journal.pone.0050171 (PMC3537678; doi:10.1371/journal.pone.0050171)
Supplement: Table S10 — Tissue samples from 63 different developmental stages in Arabidopsis. (DOCX) [file pone.0050171.s014.docx]

**Table S10 Tissue samples from 63 different developmental stages in *Arabidopsis.***

| **Sample No** | **Abbreviation** | **Tissues/organ and developmental stages** |
| --- | --- | --- |
| A1 | root 1 | root roots Wt 7 days |
| A2 | root 2 | root roots Wt 17 days |
| A3 | root 3 | root root Wt 15 days |
| A4 | root 4 | root root Wt 8 days |
| A5 | root 5 | root root Wt 8 days |
| A6 | root 6 | root root Wt 21 days |
| A7 | root 7 | root root Wt 21 days |
| A8 | hypocotyl | stem hypocotyl Wt 7 days |
| A9 | 1st node | stem 1st node Wt 21+ days |
| A10 | 2nd internode | stem stem, 2nd internode Wt 21+ days |
| A11 | cotyledons | leaf cotyledons Wt 7 days ATGE_1 |
| A12 | leaf 1 | leaf leaves 1 + 2 Wt 7 days ATGE_5 |
| A13 | leaf 2 | leaf rosette leaf #4, 1 cm long Wt 10 days |
| A14 | leaf 3 | leaf rosette leaf # 2 Wt 17 days |
| A15 | leaf 4 | leaf rosette leaf # 4 Wt 17 days |
| A16 | leaf 5 | leaf rosette leaf # 6 Wt 17 days |
| A17 | leaf 6 | leaf rosette leaf # 8 Wt 17 days |
| A18 | leaf 7 | leaf rosette leaf # 10 Wt 17 days |
| A19 | leaf 8 | leaf rosette leaf # 12 Wt 17 days |
| A20 | leaf 9 | leaf leaf 7, petiole Wt 17 days |
| A21 | leaf 10 | leaf leaf 7, proximal half Wt 17 days |
| A22 | leaf 11 | leaf leaf 7, distal half Wt 17 days |
| A23 | leaf 12 | leaf leaf Wt 15 days |
| A24 | senescing leaves | leaf senescing leaves Wt 35 days |
| A25 | cauline leaves | leaf cauline leaves Wt 21+ days |
| A26 | seedlings 1 | whole plant seedling, green parts Wt 7 days |
| A27 | seedlings 2 | whole plant seedling, green parts Wt 8 days |
| A28 | seedlings 3 | whole plant seedling, green parts Wt 8 days |
| A29 | seedlings 4 | whole plant seedling, green parts Wt 21 days |
| A30 | seedlings 5 | whole plant seedling, green parts Wt 21 days |
| A31 | whole plant 1 | whole plant developmental drift, entire rosette after transition to flowering, but before bolting Wt 21 days ATGE_22 |
| A32 | whole plant 2 | whole plant as above Wt 22 days |
| A33 | whole plant 3 | whole plant as above Wt 23 days |
| A34 | whole plant 4 | whole plant vegetative rosette Wt 7 days |
| A35 | whole plant 5 | whole plant vegetative rosette Wt 14 days |
| A36 | whole plant 6 | whole plant vegetative rosette Wt 21 days |
| A37 | shoot apex1 | apex shoot apex, vegetative + young leaves Wt 7 days |
| A38 | shoot apex2 | apex shoot apex, vegetative Wt 7 days |
| A39 | shoot apex3 | apex shoot apex, transition (before bolting) Wt 14 days |
| A40 | shoot apex4 | apex shoot apex, inflorescence (after bolting) Wt 21 days |
| A41 | flowers 1 | flowers flowers stage 9 Wt 21+ days |
| A42 | flowers 2 | flowers flowers stage 10/11 Wt 21+ days |
| A43 | flowers 3 | flowers flowers stage 12 Wt 21+ days |
| A44 | flowers 4 | flowers flowers stage 15 Wt 21+ days |
| A45 | flowers 5 | flowers flower Wt 28 days |
| A46 | pedicels | flowers stage 15, pedicels Wt 21+ days |
| A47 | sepals 1 | flowers stage 12, sepals Wt 21+ days |
| A48 | sepals 2 | flowers stage 15, sepals Wt 21+ days |
| A49 | petals 1 | flowers stage 12, petals Wt 21+ days |
| A50 | petals 2 | flowers stage 15, petals Wt 21+ days |
| A51 | stamen 1 | flowers stage 12, stamens Wt 21+ days |
| A52 | stamen 2 | flowers stage 15, stamen Wt 21+ days |
| A53 | mature pollen | mature pollen Wt 6 wk |
| A54 | carpels 1 | flowers stage 12, carpels Wt 21+ days |
| A55 | carpels 2 | flowers stage 15, carpels Wt 21+ days |
| A56 | siliques stage 3 | seeds siliques, w/ seeds stage 3; mid globular to early heart embryos Wt 8 wk |
| A57 | siliques stage 4 | seeds siliques, w/ seeds stage 4; early to late heart embryos Wt 8 wk |
| A58 | siliques stage 5 | seeds siliques, w/ seeds stage 5; late heart to mid torpedo embryos Wt 8 wk |
| A59 | seeds stage 6 | seeds seeds, stage 6, w/o siliques; mid to late torpedo embryos Wt 8 wk |
| A60 | seeds stage 7 | seeds seeds, stage 7, w/o siliques; late torpedo to early walking-stick embryos Wt 8 wk |
| A61 | seeds stage 8 | seeds seeds, stage 8, w/o siliques; walking-stick to early curled cotyledons embryos Wt 8 wk |
| A62 | seeds stage 9 | seeds seeds, stage 9, w/o siliques; curled cotyledons to early green cotyledons embryos Wt 8 wk |
| A63 | seeds stage 10 | seeds seeds, stage 10, w/o siliques; green cotyledons embryos Wt 8 wk |
